# Outcome of emergency neurosurgery in patients with refractory and super-refractory status epilepticus: a systematic review and individual participant data meta-analysis

**DOI:** 10.3389/fneur.2024.1403266

**Published:** 2024-05-28

**Authors:** Farbod Niazi, Aline Han, Lauren Stamm, Nathan A. Shlobin, Catherine Korman, Thien S. Hoang, Agnieszka Kielian, Genevieve Du Pont-Thibodeau, Laurence Ducharme Crevier, Philippe Major, Dang K. Nguyen, Alain Bouthillier, George M. Ibrahim, Aria Fallah, Aristides Hadjinicolaou, Alexander G. Weil

**Affiliations:** ^1^Department of Medicine, Université de Montréal, Montreal, QC, Canada; ^2^Brain and Development Research Axis, CHU Sainte-Justine Research Centre, Montreal, QC, Canada; ^3^Department of Medicine, McGill University, Montreal, QC, Canada; ^4^Department of Neurological Surgery, Northwestern University Feinberg School of Medicine, Chicago, IL, United States; ^5^Department of Health Sciences, Université de Montréal, Montreal, QC, Canada; ^6^Department of Neurology, Boston Children’s Hospital, Boston, MA, United States; ^7^Harvard Medical School, Boston, MA, United States; ^8^Division of Pediatric Intensive Care, Department of Pediatrics, Sainte-Justine University Hospital Centre, Montreal, QC, Canada; ^9^Division of Neurology, Department of Pediatrics, Sainte-Justine University Hospital Centre, Montreal, QC, Canada; ^10^Department of Neuroscience, Université de Montréal, Montreal, QC, Canada; ^11^Division of Neurology, University of Montreal Hospital Center (CHUM), Montreal, QC, Canada; ^12^Division of Neurosurgery, Department of Surgery, University of Montreal Hospital Center (CHUM), Montreal, QC, Canada; ^13^Division of Neurosurgery, Hospital for Sick Children, Toronto, ON, Canada; ^14^Neurosciences and Mental Health, SickKids Research Institute, Toronto, ON, Canada; ^15^Department of Neurosurgery, David Geffen School of Medicine at the University of California, Los Angeles, Los Angeles, CA, United States; ^16^Division of Neurosurgery, Department of Surgery, Sainte-Justine University Hospital Centre, Montreal, QC, Canada

**Keywords:** refractory status epilepctius (RSE), super refractory status epilepticus (SRSE), neuromodulation, epilepsy surgery, status epilepticus

## Abstract

**Background:**

Refractory (RSE) and super-refractory status epilepticus (SRSE) are serious neurological conditions requiring aggressive management. Beyond anesthetic agents, there is a lack of evidence guiding management in these patients. This systematic review and individual participant data meta-analysis (IPDMA) seeks to evaluate and compare the currently available surgical techniques for the acute treatment of RSE and SRSE.

**Methods:**

A systematic review was performed according to the Preferred Reporting Items for Systematic Reviews and Meta-Analyses for Individual Participant Data (PRISMA-IPD). Only patients who underwent surgery while in RSE and SRSE were included. Descriptive statistics were used to compare various subgroups. Multivariable logistic regression models were constructed to identify predictors of status epilepticus (SE) cessation, long-term overall seizure freedom, and favorable functional outcome (i.e., modified Rankin score of 0–2) at last follow-up.

**Results:**

A total of 87 studies including 161 participants were included. Resective surgery tended to achieve better SE cessation rate (93.9%) compared to non-resective techniques (83.9%), but this did not reach significance (*p* = 0.071). Resective techniques were also more likely to achieve seizure freedom (69.1% vs. 34.4%, *p* = <0.0001). Older age at SE (OR = 1.384[1.046–1.832], *p* = 0.023) was associated with increased likelihood of SE cessation, while longer duration of SE (OR = 0.603[0.362–1.003], *p* = 0.051) and new-onset seizures (OR = 0.244[0.069–0.860], *p* = 0.028) were associated with lower likelihood of SE cessation, but this did not reach significance for SE duration. Only shorter duration of SE prior to surgery (OR = 1.675[1.168–2.404], *p*  = 0.0060) and immediate termination of SE (OR = 3.736 [1.323–10.548], *p* = 0.014) were independently associated with long-term seizure status. Rates of favorable functional outcomes (mRS of 0–2) were comparable between resective (44.4%) and non-resective (44.1%) techniques, and no independent predictors of outcome were identified.

**Conclusion:**

Our findings suggest that emergency neurosurgery may be a safe and effective alternative in patients with RSE/SRSE and may be considered earlier during the disease course. However, the current literature is limited exclusively to small case series and case reports with high risk of publication bias. Larger clinical trials assessing long-term seizure and functional outcomes are warranted to establish robust management guidelines.

## Introduction

Status epilepticus (SE) is a serious neurological emergency characterized by abnormally prolonged seizures or recurrent seizures without return to baseline. Overall, SE has a incidence rate ranging from 9.9 to 41/100,000 per year and is associated with significant morbidity and mortality (1, 2). Current guidelines indicate the use of first-line benzodiazepines followed by appropriately selected second-line antiepileptic drugs (AEDs) (3). Refractory SE (RSE) is defined as SE not responding to first-line and appropriately selected second-line AED, and super refractory SE (SRSE) is defined as SE persisting >24 h despite the use of general anesthesia or SE that recurs after weaning of anesthetic agents (1). Beyond first and second-line treatments, there is insufficient evidence guiding management in these patients (3). Several additional options such as ketogenic diet, hypothermia, immunotherapy, and cannabidiol have been described in the literature, with various degrees of efficacy (4–7).

Achieving seizure control in a timely manner is crucial to survival and optimal recovery. In RSE and SRSE patients, treatment often requires aggressive doses of intravenous anesthetic drugs (IVADs). Prolonged IVAD use and intubation can be associated with serious complications such as infection, septic shock and increased risk of mortality (8, 9). Alternatively, surgical resection may be beneficial in a subset of patients with a clear evidence of a focal lesion and concordant semiology, imaging and electroencephalography (EEG) findings (10, 11). Recent technological advances have rendered invasive monitoring and resection safer, further enhancing their accessibility (12). However, widespread seizure networks resulting from prolonged seizures in SE may lead to suboptimal outcomes (13). Disconnective surgery, such as corpus callosotomy and multiple subpial transection (MST) have been effective at reducing seizure burden in patients with non-resectable drug-resistant epilepsy (DRE). In recent years, neurostimulation techniques, such as vagus nerve stimulation (VNS), deep brain stimulation (DBS), and responsive neurostimulation (RNS), have been increasingly adopted as a safe and effective alternative to open resective surgery in patients with DRE in whom the epileptogenic zone is widespread and cannot be localized or safely resected (e.g., eloquent cortex) (14–18). These approaches may also be a viable alternative to continued medical therapy in patients with RSE or SRSE of unknown cause with diffuse or normal imaging and EEG findings (19–21).

**Figure 1:**
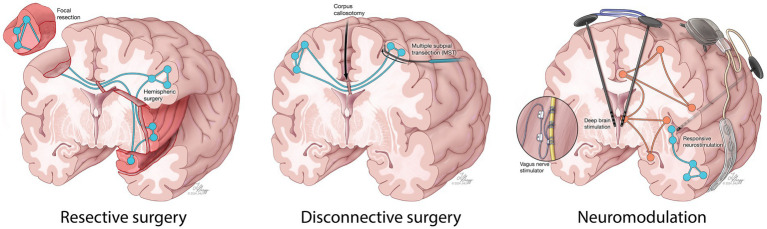
A schematic demonstration of resective, disconnective and neuromodulation surgical techniques reported in the literature for the acute treatment of RSE and SRSE.

Despite several neurosurgical approaches having been reported for the acute treatment of RSE/SRSE (Figure 1), the role of emergency neurosurgery in RSE and SRSE patients remains subject to debate with most evidence on the surgical treatment of these patients originating from case reports, or small retrospective case series. This systematic review and individual participant data meta-analysis (IPDMA) aims to gather available clinical data on all patients who underwent emergency neurosurgery for the acute control of RSE/SRSE and compare the efficacy of various neurosurgical techniques in controlling SE and subsequent seizures.

## Methods

A systematic review and individual participant data meta-analysis (IPDMA) was performed according to the Preferred Reporting Items for Systematic Reviews and Meta-Analyses of individual participant data (PRISMA-IPD) (22). The protocol for this study was developed, but not registered, prior to the conduct of this review by authors with expertise on the topic (A.H., A.F., A.G.W).

### Search strategy

We searched Medline, Embase, Cochrane Database of Systematic Reviews and Web of Science with no date or language restriction in February 2023. The search was piloted in Medline and used Medical Subject Headings (MeSH) and free-text (title, abstract, or keyword) searching to retrieve articles about neurosurgery or neuromodulation and status epilepticus. Our search strategy was developed with the help of our institution’s health sciences librarian team (T.S.H). Our full search strategy is available in Supplementary Table S1. Our search was updated in December 2023 and all relevant articles were manually added.

### Eligibility criteria

To be included in our IPDMA, the studies had to (1) be case reports, case–control or cohort studies or randomized controlled trials, (2) include patients of all age groups undergoing urgent invasive neurosurgery to abort SE, (3) include patients that were in RSE/SRSE or epilepsia partialis continua (EPC) at the time of surgery, and (4) report data on SE outcome following neurosurgical intervention. Studies were excluded if (1) they involved non-invasive therapies (e.g., transcranial magnetic stimulation, transcranial direct current stimulation), (2) they included patients that were not in SE at the time of surgery, or (3) they were conference abstracts or original studies for which full text was not available or was in a foreign language. Given that electrical status epilepticus in sleep (ESES) represents a distinct clinical entity with less urgent management as compared to acute SE, patients with ESES were excluded from our study.

### Screening and data collection

Following duplicate removal, title and abstract screening was performed by two authors (F.N., N.A.S.) to include all studies with relevance to the topic of the review. Included studies were then carefully read independently by three co-authors (F.N., A.H., L.S.) to assess eligibility according to pre-established criteria. At all stages of the screening, disagreements were resolved through discussion until consensus was reached.

Relevant data on included studies were extracted by two reviewers (F.N., N.A.S.) and stored on Microsoft Excel 2020 (Microsoft Corporation). Variables of interest included demographic data, history of epilepsy, semiology and localization of seizures, preoperative diagnostic findings, perioperative data and postoperative SE, seizure and functional outcomes. All participants were divided into two main subgroups based on type of surgical intervention: resective (i.e., focal resection with or without MST, hemispheric surgery) and non-resective (i.e., neuromodulation, open disconnective procedures). Based on a previous systematic review and meta-analysis on SRSE and a classification suggested by C.D.C., Reported etiologies were classified into six categories: known epilepsy, acute cerebral event, remote symptomatic causes, tumor, unknown and other (23).

### Outcomes

The primary outcome was cessation of SE, defined as cessation of electrographic and clinical SE. The secondary outcome was seizure freedom (Engel I or ILAE I/II) at last follow-up. For patients with known epilepsy who underwent non-resective techniques, seizure outcome was also evaluated using response to treatment (>50% reduction in seizure frequency). Finally, functional outcomes were compared using the modified Rankin Scale (mRS) (24, 25). Favorable outcome was defined as a mRS of 0–2. Given the focus on RSE/SRSE outcomes, a score of 6 was solely assigned to patients who died of causes related to their epilepsy or complications of SE and its treatment.

### Statistical analysis

Patients were stratified into various subgroups. Patient characteristics were then compared using the Chi-squared test or Fisher’s exact test for categorical variables, and Welch’s ANOVA or Mann–Whitney U test for continuous variables. All relevant variables with <40% missing values were imputed using Multiple Imputation by Chained Equations (MICE) to generate 10 datasets for each analysis (26). Given that most included studies were case reports, fixed-effect univariable and multivariable logistic regression models were constructed. All relevant predictors (*p* < 0.250 on univariable analysis) were identified. Using these variables, a fixed-effect multivariable logistic regression model was performed using forward stepwise selection of variables using Akaike information criterion (AIC). All variables that were selected in more than half of datasets were included in the final multivariable regression model. A threshold of *p* < 0.050 was selected as statistical significance and all odds ratios (ORs) are presented as OR [95% confidence interval (CI)]. For studies with more than one participant, a meta-analysis of proportions using random effects modelling and inverse variances was performed. A Freeman Tukey double arcsine transformation was applied to stabilize the variances (27). All analyses were performed on R statistical software (Rstudio version 4.3.1).

### Risk of bias assessment

Risk of bias assessment was not performed as most included studies were case reports, and thus, had an inherently high risk of bias.

## Results

**Figure 2:**
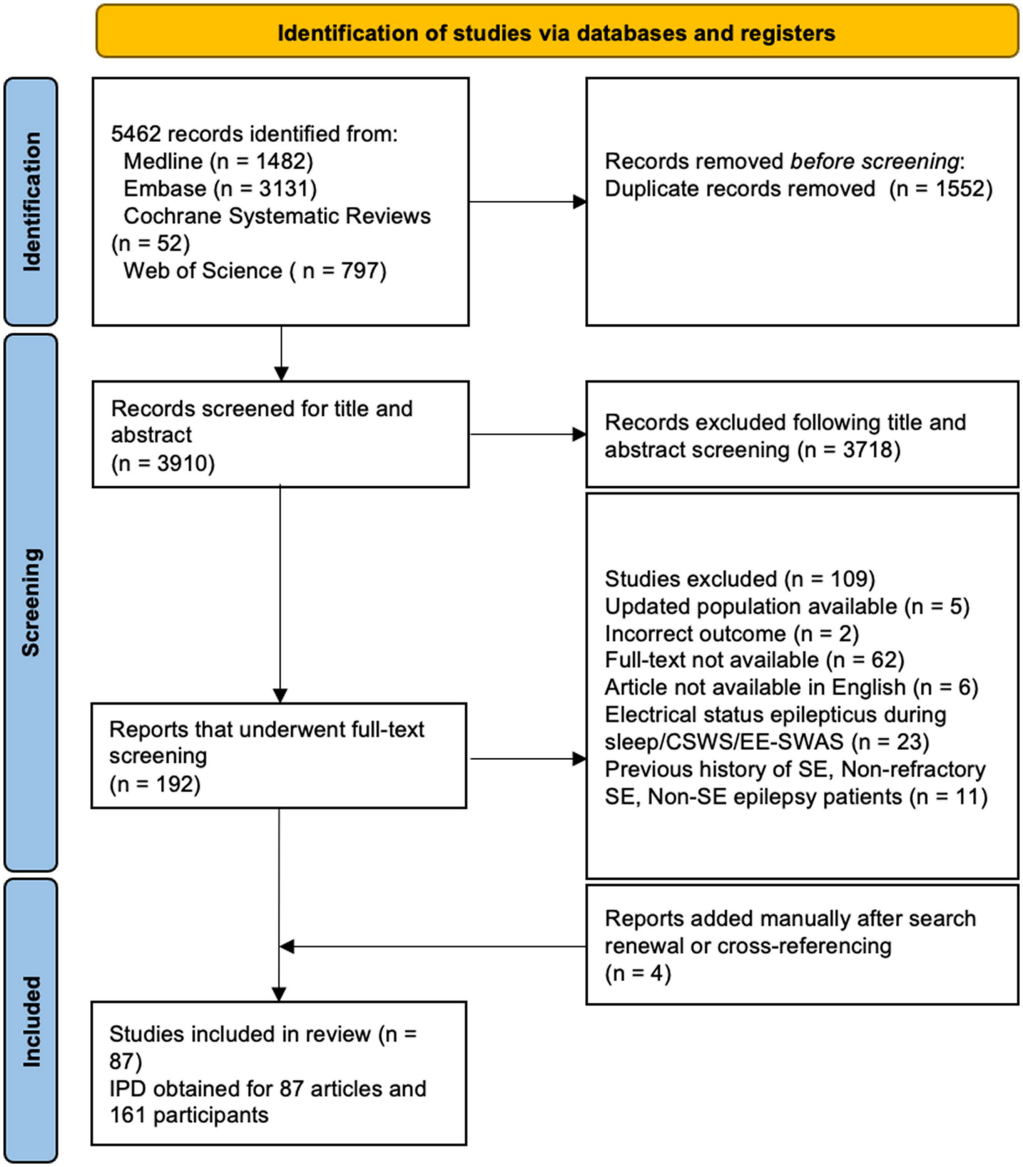
PRIMSA flowchart.

Our search yielded 3,910 articles after deduplication, of which 83 met our eligibility criteria. An additional four articles were manually added after search renewal, resulting in the inclusion of 87 articles in our final analysis. The characteristics of included studies are available in Supplementary Table S2. The PRIMSA flowchart demonstrating the inclusion of articles is available in Figure 2. In total, 66 case reports on 66 patients and 21 retrospective case series on 95 patients including IPD on a total of 161 patients were included in our analysis. IPD was provided in all studies, and no issues regarding IPD were identified. Clinical characteristics of all patients in this study can be found in Table 1. Given that most included studies were case reports and small, retrospective case series, the risk of bias was considered high in all studies.

**Table 1:** Clinical characteristics of all included patients.

Characteristic	Entire cohort (*N* = 161)	*N*
Sex (F)	61 (46.6%)	131
Age at SE onset (years)		160
Median (IQR)	14.5 (5.6–26.0)	
Mean (SD)	19.2 (18.2)	
Range	0.0–68.0	
New-onset seizures or epilepsy	42 (26.1%)	161
History of SE	21 (17.4%)	121
SRSE	136 (88.3%)	154
Time in SE prior to surgery (days)		132
Median (IQR)	22.5 (13.8–42.0)	
Mean (SD)	110.6 (397.3)	
Range	2.0–3468.0	
SE localization based on EEG findings		159
Focal	127 (79.9%)	
Generalized	32 (20.1%)	
SE semiology classification		159
FTBTC SE	36 (22.6%)	
FIAS SE	40 (25.2%)	
Focal aware SE	54 (34.0%)	
Generalized NCSE	8 (5.0%)	
Generalized myoclonic SE	4 (2.5%)	
Generalized convulsive SE	17 (10.7%)	
AED use		
Levetiracetam	60 (54.1%)	111
Phenytoin	61 (55.5%)	110
Fosphenytoin	16 (14.5%)	110
Topiramate	35 (31.8%)	110
Valproic Acid	51 (45.9%)	111
Phenobarbital	65 (52.0%)	125
IVAD use		
Midazolam	81 (64.8%)	125
Ketamine	25 (20.0%)	125
Propofol	43 (34.4%)	125
Thiopental	15 (12.0%)	125
Pentobarbital	32 (25.6%)	125
Burst suppression	62 (52.1%)	119
Focal slowing	14 (10.6%)	132
Diffuse slowing	19 (14.4%)	132
MRI findings		135
Focal	75 (55.6%)	
Diffuse	34 (25.2%)	
Negative	26 (19.3%)	
Concordant MRI/EEG findings	57 (46.0%)	124
SE cessation after intervention	145 (90.1%)	161
Immediate SE cessation	63 (57.8%)	109
Time to SE cessation (days)		109
Median (IQR)	0.0 (0.0–7.0)	
Mean (SD)	7.4 (14.2)	
Range	0.0–76.0	
Reoperation	20 (12.4%)	161
Indication for reoperation		20
Seizure control	10 (50.0%)	
SE control	10 (50.0%)	
Seizure freedom	88 (55.7%)	158
Surgical approach		161
Non-resective	62 (38.5%)	
Resective	99 (61.5%)	
Surgical treatment		161
Focal resection	69 (42.9%)	
Hemispheric surgery	26 (16.1%)	
MST +	4 (2.5%)	
MST	5 (3.1%)	
Corpus callosotomy	5 (3.1%)	
VNS	32 (19.9%)	
DBS	10 (6.2%)	
RNS	4 (2.5%)	
SCS	6 (3.7%)	
Etiology		153
Known epilepsy	80 (52.3%)	
Remote symptomatic	5 (3.3%)	
Unknown	44 (28.8%)	
Acute cerebral event	15 (9.8%)	
Tumor	6 (3.9%)	
Other	3 (2.0%)	
mRS		79
0	13 (16.5%)	
1	12 (15.2%)	
2	9 (11.4%)	
3	13 (16.5%)	
4	18 (22.8%)	
5	5 (6.3%)	
6	9 (11.4%)	
Favorable mRS (0–2)	35 (44.3%)	79
Death	14 (8.7%)	161
Epilepsy/SE-related death	9 (5.6%)	161

SE, status epilepticus; FTBTC, focal to bilateral tonic–clonic; FIAS, focal impaired awareness; NCSE, non-convulsive status epilepticus; MRI, magnetic resonance imaging; EEG, electroencephalogram; mRS, modified Rankin Scale; RSE, refractory status epilepticus; SRSE, super-refractory status epilepticus; RNS, responsive neurostimulation; VNS, vagus nerve stimulation; DBS, deep brain stimulation; MST, multiple subpial transection; SCS, subdural cortical stimulation.

### Patient characteristics

**Figure 3:**
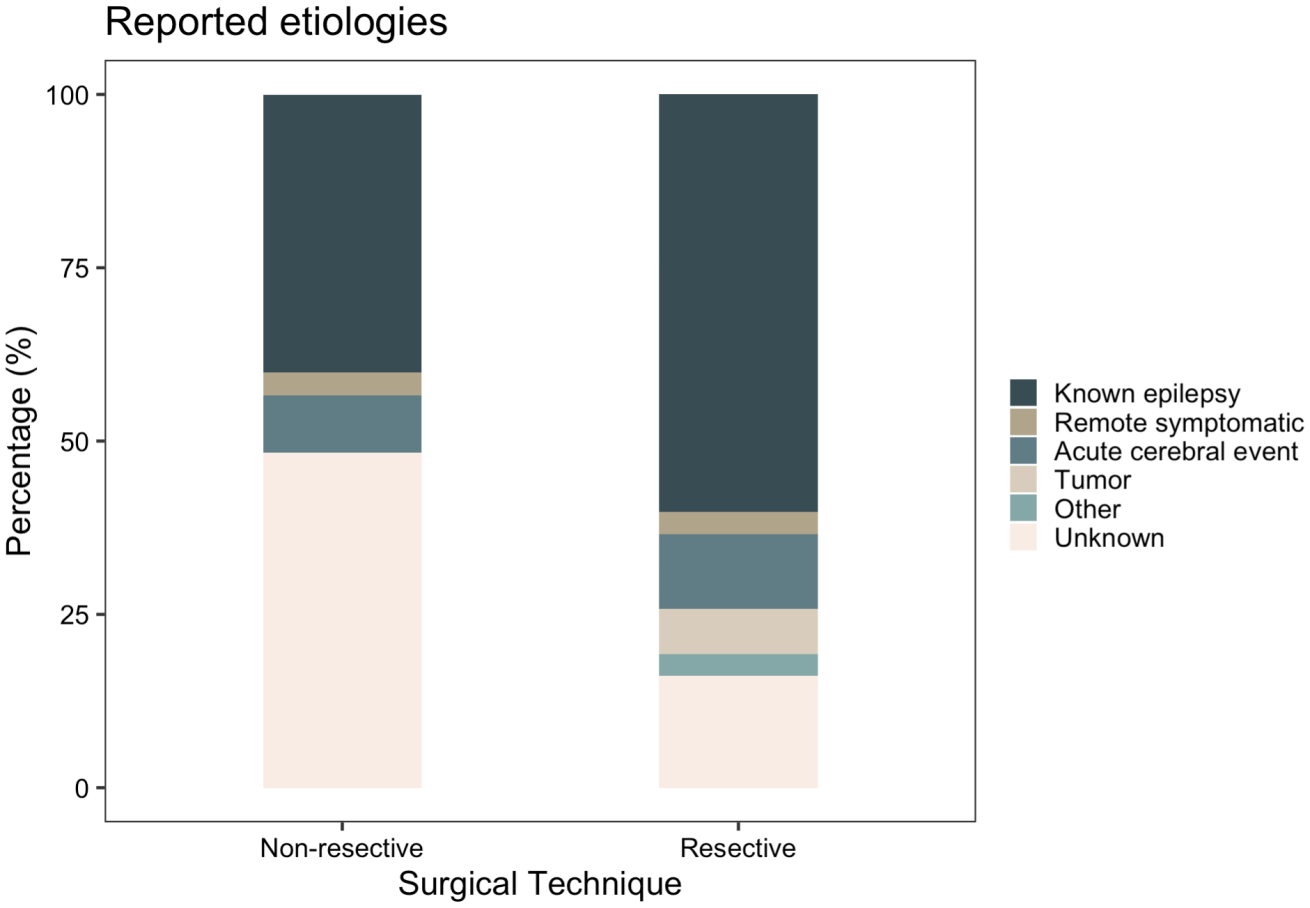
Reported etiology of SE in the resective and non-resective subgroups.

Clinical characteristics of patients stratified by surgical technique and age are displayed in Tables 2 and 3, respectively. Supplementary Tables S3–S5 included patient characteristics stratified by SE outcome, mRS and death, respectively. Mean (SD) age at SE was 19.2 (SD: 18.2, range: 0–68) years and was not significantly different between the resective and non-resective subgroups (*p* = 0.27). Overall, 26.1% of patients presented with new-onset seizures that led to SE. Adult patients were more likely to have new-onset seizures, compared to pediatric patients (*p* = 0.0005). The most commonly used AEDs were phenytoin (55.5%), levetiracetam (54.1%) and phenobarbital (52.0%). Most patients (88.3%) were in SRSE at the time of surgery and 30 (18.6%) were in EPC. Some degree of induced burst suppression was achieved in 52.1% of patients. The most commonly used IVADs were midazolam (64.8%), propofol (34.4%) and pentobarbital (25.6%). Median time spent in SE prior to surgery was 22.5 days. On average, duration of SE prior to surgery was longer in the non-resective subgroup (*p* = 0.0014), but this was highly variable across studies with some patients having EPC for as long as 9.5 years. The most common semiology was focal aware SE (34.0%) including focal motor SE and EPC. The most common etiology among all patients was various types of malformations of cortical development (MCD) present in 33.1% of all patients. Most patients (60.2%) in the resective subgroup had a known history of epilepsy leading to SE, while the most common etiology in the non-resective cohort was unknown SE (48.3%; Figure 3). Among patients with EPC, the most common cause was EPC of unknown etiology in 26.7%, followed by Rasmussen’s encephalitis in 23.3%.

**Table 2:** Clinical characteristics and postoperative outcome of patients included in the study stratified by surgical technique.

Characteristic	Non-resective		Resective		*p*-value
	*N* = 62	*N*	*N* = 99	*N*	
Sex (F)	29 (49.2%)	59	32 (44.4%)	72	0.72
Age at SE onset (years)		62		98	0.27
Median (IQR)	17.0 (6.0–25.8)		10.5 (5.0–26.3)		
Mean (SD)	19.0 (15.5)		19.2 (19.8)		
Range	0.5–67.0		0.0–68.0		
New-onset seizures	21 (33.9%)	62	21 (21.2%)	99	0.11
History of SE	15 (25.4%)	59	6 (9.7%)	62	0.041^*^
SRSE	53 (89.8%)	59	83 (87.4%)	95	0.84
Time in SE prior to surgery (days)		49		83	0.0014^*^
Median (IQR)	30.0 (19.0–80.0)		20.0 (11.5–33.5)		
Mean (SD)	214.6 (607.0)		49.2 (161.6)		
Range	5.0–3468.0		2.0–1460.0		
EEG Localization		60		99	<0.0001^*^
Focal	28 (46.7%)		99 (100.0%)		
Generalized	32 (53.3%)		0 (0.0%)		
SE semiology classification		60		99	0.005^*^
FTBTC SE	9 (15.0%)		27 (27.3%)		
FIAS SE	4 (6.7%)		36 (36.4%)		
Focal aware SE	18 (30.0%)		36 (36.4%)		
Generalized NCSE	8 (13.3%)		0 (0.0%)		
Generalized myoclonic SE	4 (6.7%)		0 (0.0%)		
Generalized convulsive SE	17 (28.3%)		0 (0.0%)		
Focal slowing	3 (6.4%)	47	11 (12.9%)	85	0.37
Diffuse slowing	11 (23.4%)	47	8 (9.4%)	85	0.053
MRI finding		44		91	<0.0001^*^
Focal	8 (18.2%)		67 (73.6%)		
Diffuse	15 (34.1%)		19 (20.9%)		
Negative	21 (47.7%)		5 (5.5%)		
Concordant MRI-EEG findings	4 (9.1%)	44	53 (66.3%)	80	<0.001^*^
Etiology		60		93	0.0005^*^
Known epilepsy	24 (40.0%)		56 (60.2%)		
Remote symptomatic	2 (3.3%)		3 (3.2%)		
Unknown	29 (48.3%)		15 (16.1%)		
Acute cerebral event	5 (8.3%)		10 (10.8%)		
Tumor	0 (0.0%)		6 (6.5%)		
Other	0 (0.0%)		3 (3.2%)		
SE cessation after initial intervention	52 (83.9%)	62	93 (93.9%)	99	0.071
Time to SE cessation (days)		48		61	<0.001^*^
Median (IQR)	7.0 (3.0–18.0)		0.0 (0.0–0.0)		
Mean (SD)	11.9 (12.8)		3.8 (14.3)		
Range	0.0–60.0		0.0–76.0		
Immediate SE cessation	11 (22.0%)	48	55 (88.7%)	61	<0.0001^*^
Reoperation	10 (16.1%)	62	10 (10.1%)	99	0.38
Indication for reoperation^b^		10		10	0.66
SE control	4 (40.0%)		6 (60.0%)		
Seizure control	6 (60.0%)		4 (40.0%)		
Seizure freedom	21 (34.4%)	61	7 (69.1%)	97	<0.0001^*^
mRS		34		45	0.81
0	6 (17.6%)		7 (15.6%)		
1	4 (11.8%)		8 (17.8%)		
2	4 (11.8%)		5 (11.1%)		
3	5 (14.7%)		8 (17.8%)		
4	7 (20.6%)		11 (24.4%)		
5	3 (8.8%)		2 (4.4%)		
6	5 (14.7%)		4 (8.9%)		
Favorable mRS (0–2)	15 (44.1%)	34	20 (44.4%)	45	>0.99
Death	8 (12.9%)	62	6 (6.1%)	99	0.20
Epilepsy/SE-related death	5 (8.1%)	62	4 (4.0%)	99	0.47

SE, status epilepticus; FTBTC, focal to bilateral tonic–clonic; FIAS, focal impaired awareness; NCSE, non-convulsive status epilepticus; MRI, magnetic resonance imaging; EEG, electroencephalogram; mRS, modified Rankin Scale. All categorical variables are displayed in frequency (valid percentage) unless otherwise specified. ^a^Chi-squared test or Fisher exact test for categorical variables and Welch’s ANOVA or Mann–Whitney U test for numeric variables. ^b^Relative frequency is calculated based on patients who underwent reoperation. ^*^Statistically significant.

**Table 3:** Clinical characteristics and outcome of included patients stratified by age group.

Characteristic	Adult (>18 years old)		Pediatric (<18 years old)		*p*-value
	*N* = 65	*N*	*N* = 95	*N*	
Sex (F)	27 (42.9%)	63	34 (50.7%)	67	0.47
New-onset seizures	27 (41.5%)	65	15 (15.8%)	95	0.0005^*^
History of SE	7 (13.0%)	54	14 (21.2%)	66	0.35
SRSE	55 (88.7%)	62	81 (89.0%)	91	>0.99
Time in SE prior to surgery (days)		49		82	0.81
Median (IQR)	21.0 (13.0–42.0)		23.5 (14.0–42.0)		
Mean (SD)	99.7 (290.9)		116.2 (452.6)		
Range	3.0–1,460.0		2.0–3,468.0		
EEG Localization		65		93	0.89
Focal	51 (78.5%)		75 (80.6%)		
Generalized	14 (21.5%)		18 (19.4%)		
SE semiology classification		65		93	0.70
FTBTC SE	16 (24.6%)		20 (21.5%)		
FIAS SE	19 (29.2%)		21 (22.6%)		
Focal aware SE	17 (26.2%)		36 (38.7%)		
Generalized NCSE	3 (4.6%)		5 (5.4%)		
Generalized myoclonic SE	2 (3.1%)		2 (2.2%)		
Generalized convulsive SE	8 (12.3%)		9 (9.7%)		
Focal slowing	5 (9.6%)	52	8 (10.1%)	79	>0.99
Diffuse slowing	9 (17.3%)	52	10 (12.7%)	79	0.63
MRI finding		57		77	0.86
Focal	30 (52.6%)		44 (57.1%)		
Diffuse	15 (26.3%)		19 (24.7%)		
Negative	12 (21.1%)		14 (18.2%)		
Concordant MRI-EEG findings	21 (43.8%)	48	35 (46.7%)	75	0.90
Etiology		65		87	0.0005^*^
Known epilepsy	19 (29.2%)		60 (69.0%)		
Remote symptomatic	3 (4.6%)		2 (2.3%)		
Unknown	24 (36.9%)		20 (23.0%)		
Acute cerebral event	14 (21.5%)		1 (1.1%)		
Tumor	5 (7.7%)		1 (1.1%)		
Other	0 (0.0%)		3 (3.4%)		
Surgical technique		65		95	0.25
Neuromodulation	25 (38.5%)		27 (28.4%)		
Open surgery	40 (61.5%)		68 (71.6%)		
Goal of surgery		65		95	0.27
Non-resective	29 (44.6%)		33 (34.7%)		
Resective	36 (55.4%)		62 (65.3%)		
SE cessation after intervention	60 (92.3%)	65	84 (88.4%)	95	0.59
Immediate SE cessation	30 (55.6%)	54	32 (59.3%)	54	0.85
Time to SE cessation (days)		54		54	>0.99
Median (IQR)	0.0 (0.0–6.5)		0.0 (0.0–10.0)		
Mean (SD)	7.4 (15.4)		7.5 (13.1)		
Range	0.0–76.0		0.0–60.0		
Reoperation	5 (7.7%)	65	15 (15.8%)	95	0.20
Indication for reoperation^b^		5		15	>0.99
Seizure control	2 (40.0%)		8 (53.3%)		
SE control	3 (60.0%)		7 (46.7%)		
Seizure freedom	36 (57.1%)	63	52 (55.3%)	94	0.95
Response to treatment (only disconnective surgery)	12 (75.0%)	16	14 (73.7%)	19	>0.99
Favorable mRS	22 (47.8%)	46	12 (37.5%)	32	0.50
Epilepsy/SE-related death	7 (10.8%)	65	2 (2.1%)	95	0.047^*^
Death	8 (12.3%)	65	6 (6.3%)	95	0.30

SE, status epilepticus; FTBTC, focal to bilateral tonic–clonic; FIAS, focal impaired awareness; NCSE, non-convulsive status epilepticus; MRI, magnetic resonance imaging; EEG, electroencephalogram; mRS, modified Rankin Scale. All categorical variables are displayed in frequency (valid percentage) unless otherwise specified. ^a^Chi-squared test or Fisher exact test for categorical variables and Welch’s ANOVA or Mann–Whitney U test for numeric variables. ^b^Relative frequency is calculated based on patients who underwent reoperation. ^*^Statistically significant.

### Surgical interventions

The most common surgical technique was focal resection performed in 69 patients (42.9%). A breakdown of resective techniques and their outcomes is available in Table 4 and Supplementary Table S6. Among the 62 patients who underwent non-resective surgery, 10 (16.1%) underwent open disconnection: 5 MST and 5 corpus callosotomy. Overall, 52 patients underwent neuromodulation (Table 5). The most common neuromodulation technique was VNS, which was performed in 32 patients. Stimulation parameters, as well as titration periods were highly variable across studies (Supplementary Table S7). The average titration period was 8.9 days (SD: 11.5). Output current at onset of VNS was mostly 0.25 mA. Output current was increased at rates of 0.25–0.75 mA/24 h to obtain a final output varying between 0.25 to 3 mA. When reported, side effects included coughing and bradycardia. DBS was performed in 10. For DBS, the most common targets were thalamic nuclei: including anterior thalamic nucleus (ANT) in three and centromedian nucleus (CMN) in five cases. Two patients with EPC underwent DBS of unconventional motor targets. One patient had an unknown etiology and underwent unilateral stimulation of the globus pallidus internus (GPi), while the other had Rasmussen’s encephalitis and underwent unilateral stimulation of the caudal zona incerta (CZi), since he was not considered an ideal candidate for hemispheric surgery. Four patients underwent RNS, of which three had at least one active lead placed on the precentral gyrus, and one had both depth leads in the occipital lobe. Of the four patients who underwent RNS, one patient had preoperative investigation with stereo-electroencephalography (SEEG) only while one was investigated with both extraoperative electrocorticography (ECoG) and SEEG. One patient had intraoperative ECoG and one patient did not undergo invasive preoperative or intraoperative monitoring.

**Table 4:** Clinical characteristics and postoperative outcome of patients who underwent open resective surgery stratified by surgical technique.

Characteristic	Focal resection		Hemispheric surgery		MST +		*p*-value
	*N* = 69	*N*	*N* = 26	*N*	*N* = 4	*N*	
Sex (F)	22 (44.0%)	50	9 (50.0%)	18	1 (25.0%)	4	0.72
Age at SE onset (years)		68		26		4	0.046^*^
Median (IQR)	13.0 (6.0–34.0)		7.0 (1.3–15.5)		29.0 (18.0–39.0)	4	
Mean (SD)	21.8 (21.3)		11.3 (13.3)		28.0 (18.1)		
Range	0.0–68.0		0.1–56.0		6.0–48.0		
New-onset seizures	20 (29.0%)	69	1 (3.8%)	26	0 (0.0%)	4	0.022^*^
History of SE	4 (9.8%)	41	1 (5.9%)	17	1 (25.0%)	4	0.55
SRSE	57 (86.4%)	66	23 (92.0%)	25	3 (75.0%)	4	0.55
Time in SE prior to surgery (days)		57		22		4	0.50
Median (IQR)	21.0 (14.0–33.0)		11.0 (5.3–30.0)		35.0 (25.0–45.5)		
Mean (SD)	36.6 (44.9)		84.4 (307.9)		35.5 (21.6)		
Range	2.0–183.0		3.0–1,460.0		10.0–62.0		
EEG localization		69		26		4	NA
Focal	69 (100.0%)		26 (100.0%)		4 (100.0%)		
Generalized	0 (0.0%)		0 (0.0%)		0 (0.0%)		
SE semiology classification		69		26		4	0.016^*^
FTBTC SE	22 (31.9%)		4 (15.4%)		1 (25.0%)		
FIAS SE	29 (42.0%)		6 (23.1%)		1 (25.0%)		
Focal aware SE	18 (26.1%)		16 (61.5%)		2 (50.0%)		
Generalized NCSE	0 (0.0%)		0 (0.0%)		0 (0.0%)		
Generalized myoclonic SE	0 (0.0%)		0 (0.0%)		0 (0.0%)		
Generalized convulsive SE	0 (0.0%)		0 (0.0%)		0 (0.0%)		
Focal slowing	6 (10.7%)	56	4 (16.0%)	25	1 (25.0%)	4	0.61
Diffuse slowing	4 (7.1%)	56	2 (8.0%)	25	2 (50.0%)	4	0.053
MRI finding		64		23		4	0.20
Focal	43 (67.2%)		21 (91.3%)		3 (75.0%)		
Diffuse	16 (25.0%)		2 (8.7%)		1 (25.0%)		
Negative	5 (7.8%)		0 (0.0%)		0 (0.0%)		
Concordant MRI-EEG findings	36 (66.7%)	54	16 (72.7%)	22	1 (25.0%)	4	0.22
SE cessation after intervention	63 (91.3%)	69	26 (100.0%)	26	4 (100.0%)	4	0.27
Time to SE cessation (days)		46		12		4	>0.99
Median (IQR)	0.0 (0.0–0.0)		0.0 (0.0–0.0)		0.0 (0.0–0.0)		
Mean (SD)	5.1 (16.3)		0.0 (0.0)		0.0 (0.0)		
Range	0.0–76.0		0.0–0.0		0.0–0.0		
Immediate SE cessation	40 (85.1%)	46	12 (100.0%)	12	3 (100.0%)	3	0.28
Reoperation	9 (13.0%)	69	1 (3.8%)	26	0 (0.0%)	3	0.33
Indication for reoperation^b^		9		1		0	>0.99
SE control	4 (44.4%)		0 (0.0%)		N/A		
Seizure control	5 (55.6%)		1 (100.0%)		N/A		
Seizure freedom	42 (62.7%)	67	22 (84.6%)	26	3 (75.0%)	4	0.13
Favorable mRS	17 (45.9%)	37	2 (28.6%)	7	1 (100.0%)	1	0.32
Death	5 (7.2%)	69	1 (3.8%)	26	0 (0.0%)	4	0.75
Epilepsy/SE-related death	4 (5.8%)	69	0 (0.0%)	26	0 (0.0%)	4	0.40

MST, multiple subpial transection; SE, status epilepticus; FTBTC, focal to bilateral tonic–clonic; FIAS, focal impaired awareness; NCSE, non-convulsive status epilepticus; MRI, magnetic resonance imaging; EEG, electroencephalogram; mRS, modified Rankin Scale; N/A, not applicable. All categorical variables are displayed in frequency (valid percentage) unless otherwise specified. ^a^Chi-squared test or Fisher exact test for categorical variables and Welch’s ANOVA or Mann–Whitney U test for numeric variables. ^b^Relative frequency is calculated based on patients who underwent reoperation. ^*^Statistically significant.

**Table 5:** Clinical characteristics and postoperative outcome of patients who underwent disconnective surgery stratified by surgical technique.

	Open disconnective surgery				Neuromodulation								
Characteristic	MST		Corpus callosotomy		VNS		DBS		SCS		RNS		*p*-value
	*N* = 5	N	*N* = 5	*N*	*N* = 32	*N*	*N* = 10	*N*	*N* = 6	*N*	*N* = 4	*N*	
Sex (F)	1 (33.3%)	3	2 (40.0%)	5	18 (56.3%)	32	5 (50.0%)	10	2 (33.3%)	6	1 (33.3%)	3	0.87
Age at SE onset (years)		5		5		32		10		6		4	0.47
Median (IQR)	5.0 (4.0–17.0)		24.0 (16.0–25.0)		13.5 (2.2–26.3)		19.5 (15.5–26.5)		23.0 (21.0–25.8)		24.0 (21.5–28.8)		
Mean (SD)	9.3 (8.1)		21.0 (8.6)		17.2 (18.1)		23.5 (17.0)		23.2 (2.8)		26.3 (7.6)		
Range	1.6–19.0		9.0–31.0		0.5–67.0		5.0–66.0		20.0–26.0		20.0–37.0		
New-onset seizures	0 (0.0%)	5	1 (20.0%)	5	14 (43.8%)	32	4 (40.0%)	10	1 (16.7%)	6	1 (25.0%)	4	0.40
History of SE	0 (0.0%)	3	1 (20.0%)	5	8 (25.8%)	31	4 (40.0%)	10	0 (0.0%)	6	2 (50.0%)	4	0.35
SRSE	5 (100.0%)	5	5 (100.0%)	5	28 (87.5%)	32	8 (80.0%)	10	3 (100.0%)	3	4 (100.0%)	4	0.70
Time in SE prior to surgery (days)		2		5		30		8		1		3	0.98
Median (IQR)	94.0 (57.0–131.0)		42.0 (30.0–90.0)		28.0 (15.3–66.8)		36.0 (27.8–61.3)		90.0 (90.0–90.0)		17.0 (15.0–18.5)		
Mean (SD)	94.0 (104.7)		121.0 (169.2)		261.4 (731.7)		217.6 (502.4)		90.0 (NA)		16.7 (3.5)		
Range	20.0–168.0		23.0–420.0		5.0–3,468.0		19.0–1,460.0		90.0–90.0		13.0–20.0		
EEG localization		5		5		30		10		6		4	0.0010^*^
Focal	5 (100.0%)		2 (40.0%)		8 (26.7%)		4 (40.0%)		5 (83.3%)		4 (100.0%)		
Generalized	0 (0.0%)		3 (60.0%)		22 (73.3%)		6 (60.0%)		1 (16.7%)		0 (0.0%)		
SE semiology classification		5		5		30		10		6		4	0.46
FTBTC SE	2 (40.0%)		1 (20.0%)		3 (10.0%)		2 (20.0%)		0 (0.0%)		1 (25.0%)		
FIAS SE	1 (20.0%)		1 (20.0%)		1 (3.3%)		0 (0.0%)		1 (16.7%)		0 (0.0%)		
Focal aware SE	2 (40.0%)		0 (0.0%)		7 (23.3%)		2 (20.0%)		4 (66.7%)		3 (75.0%)		
Generalized NCSE	0 (0.0%)		1 (20.0%)		5 (16.7%)		2 (20.0%)		0 (0.0%)		0 (0.0%)		
Generalized myoclonic SE	0 (0.0%)		0 (0.0%)		3 (10.0%)		1 (10.0%)		0 (0.0%)		0 (0.0%)		
Generalized convulsive SE	0 (0.0%)		2 (40.0%)		11 (36.7%)		3 (30.0%)		1 (16.7%)		0 (0.0%)		
Focal slowing	0 (0.0%)	4	0 (0.0%)	5	2 (9.1%)	22	0 (0.0%)	6	1 (16.7%)	6	0 (0.0%)	4	>0.99
Diffuse slowing	0 (0.0%)	4	3 (60.0%)	5	5 (22.7%)	22	3 (50.0%)	6	0 (0.0%)	6	0 (0.0%)	4	0.051
MRI finding		5		4		17		8		6		4	0.076
Focal	0 (0.0%)		1 (25.0%)		2 (11.8%)		1 (12.5%)		1 (16.7%)		3 (75.0%)		
Diffuse	0 (0.0%)		2 (50.0%)		7 (41.2%)		4 (50.0%)		2 (33.3%)		0 (0.0%)		
Negative	5 (100.0%)		1 (25.0%)		8 (47.1%)		3 (37.5%)		3 (50.0%)		1 (25.0%)		
Concordant MRI-EEG findings	0 (0.0%)	5	1 (25.0%)	4	0 (0.0%)	17	0 (0.0%)	8	0 (0.0%)	6	3 (75.0%)	4	0.0005^*^
SE cessation after intervention	2 (40.0%)	5	4 (80.0%)	5	29 (90.6%)	32	8 (80.0%)	10	5 (83.3%)	6	4 (100.0%)	4	0.10
Time to SE cessation (days)		2		2		28		7		5		4	
Median (IQR)	0.0 (0.0–0.0)		0.0 (0.0–0.0)		8.5 (4.8–20.0)		4.0 (2.5–18.0)		0.0 (0.0–4.0)		17.5 (14.8–20.3)		
Mean (SD)	0.0 (0.0)		0.0 (0.0)		14.8 (14.0)		11.1 (12.3)		1.8 (2.5)		17.5 (3.5)		
Range	0.0–0.0		0.0–0.0		0.0–60.0		0.0–33.0		0.0–5.0		14.0–21.0		
Immediate SE cessation	2 (100.0%)	2	2 (100.0%)	2	1 (3.6%)	28	1 (14.3%)	7	3 (60.0%)	5	0 (0.0%)	4	<0.0005
Reoperation	4 (80.0%)	5	2 (40.0%)	5	2 (6.3%)	32	1 (10.0%)	10	1 (16.7%)	6	0 (0.0%)	4	0.0010^*^
Indication for reoperation^b^		4		2		2		1		1		0	0.61
SE control	3 (75.0%)		1 (50.0%)		1 (50.0%)		0 (0.0%)		0 (0.0%)		N/A		
Seizure control	1 (25.0%)		1 (50.0%)		1 (50.0%)		1 (100.0%)		1 (100.0%)		N/A		
Seizure freedom	4 (80.0%)	5	2 (40.0%)	5	12 (38.7%)	31	2 (20.0%)	10	1 (16.7%)	6	0 (0.0%)	4	0.12
Response to treatment^c^	1 (100.0%)	1	2 (66.7%)	3	14 (77.8%)	18	2 (40.0%)	5	5 (100.0%)	5	2 (66.7%)	3	0.36
Favorable mRS	2 (66.7%)	3	1 (33.3%)	3	7 (38.9%)	18	1 (25.0%)	4	2 (66.7%)	3	2 (66.7%)	3	0.79
Death	0 (0.0%)	5	0 (0.0%)	5	7 (21.9%)	32	0 (0.0%)	10	1 (16.7%)	6	0 (0.0%)	4	0.28
Epilepsy/SE-related death	0 (0.0%)	5	0 (0.0%)	5	4 (12.5%)	32	0 (0.0%)	10	1 (16.7%)	6	0 (0.0%)	4	0.62

MST, multiple subpial transection; VNS, vagus nerve stimulation; DBS, deep brain stimulation; SCS, subdural cortical stimulation; RNS, responsive neurostimulation; SE, status epilepticus; FTBTC, focal to bilateral tonico-clonic; FIAS, focal impaired awareness; NCSE, non-convulsive status epilepticus; MRI, magnetic resonance imaging; EEG, electroencephalogram; mRS, modified Rankin Scale; N/A, not applicable. All categorical variables are displayed in frequency (valid percentage) unless otherwise specified. ^a^Chi-squared test or Fisher exact test for categorical variables and Welch’s ANOVA or Mann–Whitney U test for numeric variables. ^b^Relative frequency is calculated based on patients who underwent reoperation. ^c^Only patients with prior epilepsy who underwent only non-resective surgery included. ^*^Statistically significant.

### Patient outcomes

**Figure 4:**
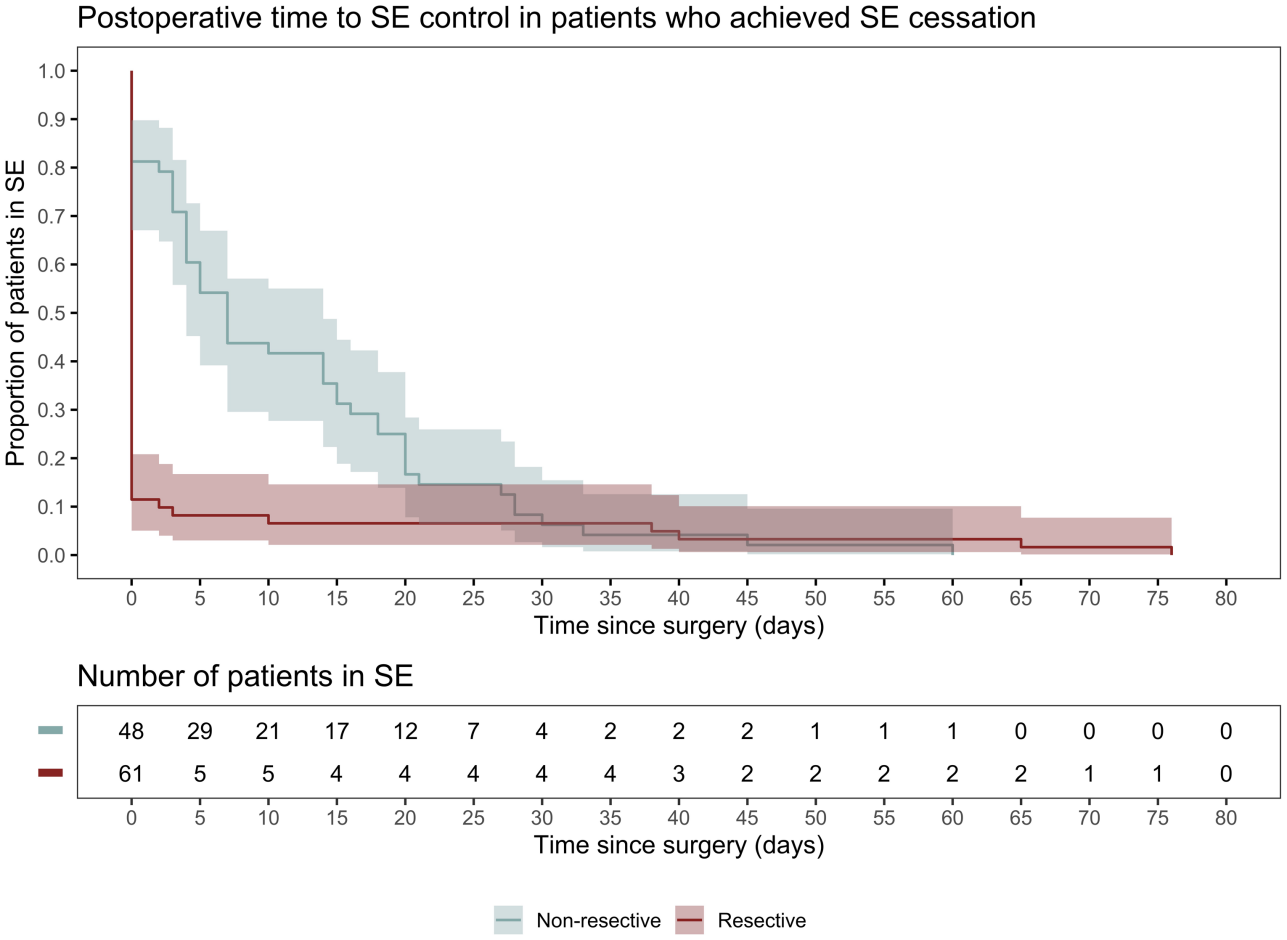
Kaplan–Meier curve demonstrating time spent in SE following surgery only in patients who achieved SE cessation following surgical intervention.

**Figure 5:**
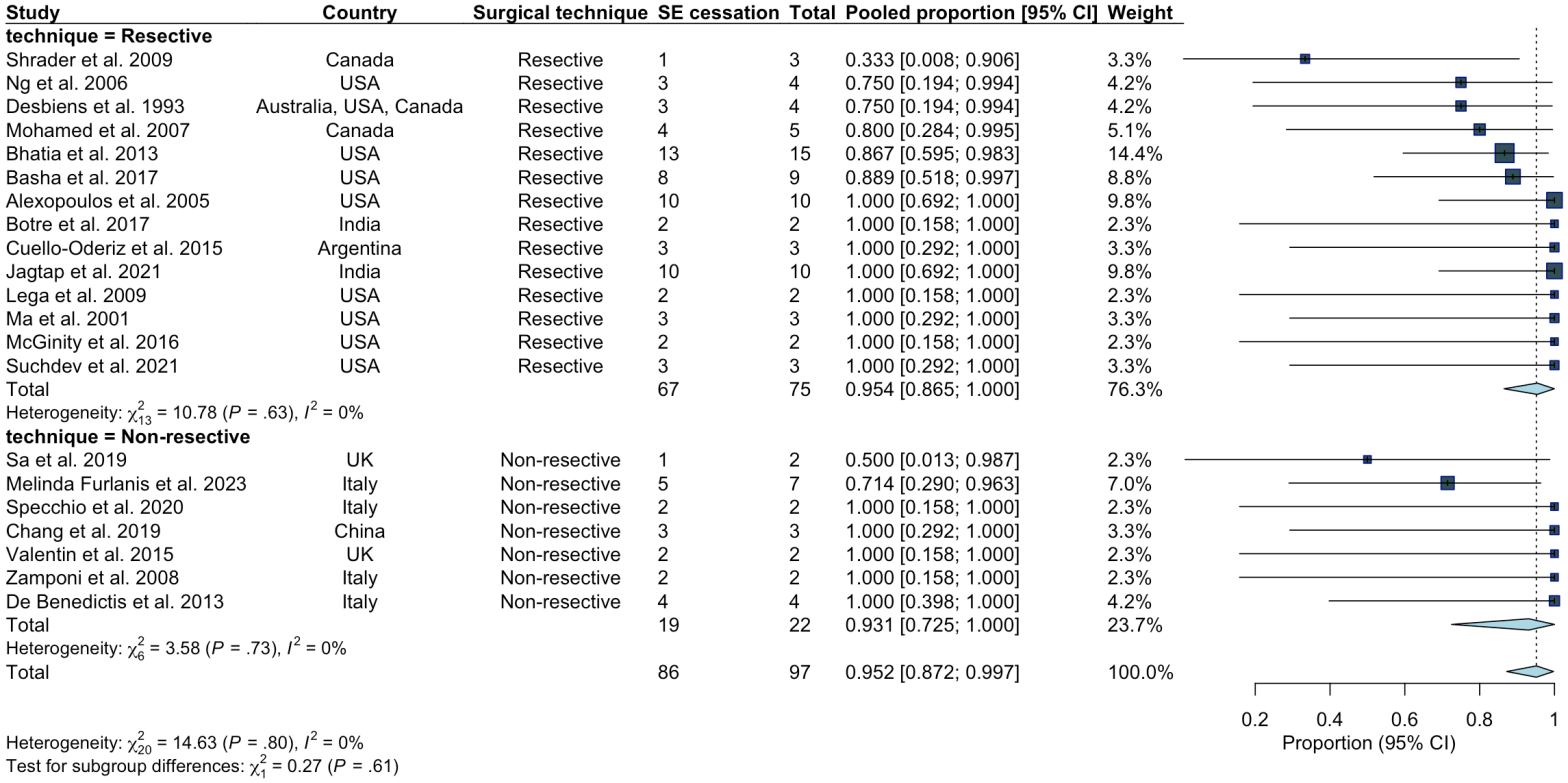
Pooled proportion of overall rate of SE cessation in all studies including more than one participant. Random effects modeling with inverse variance was used. A Freeman Tukey double arcsine transformation was applied to stabilize the variances.

**Figure 6:**
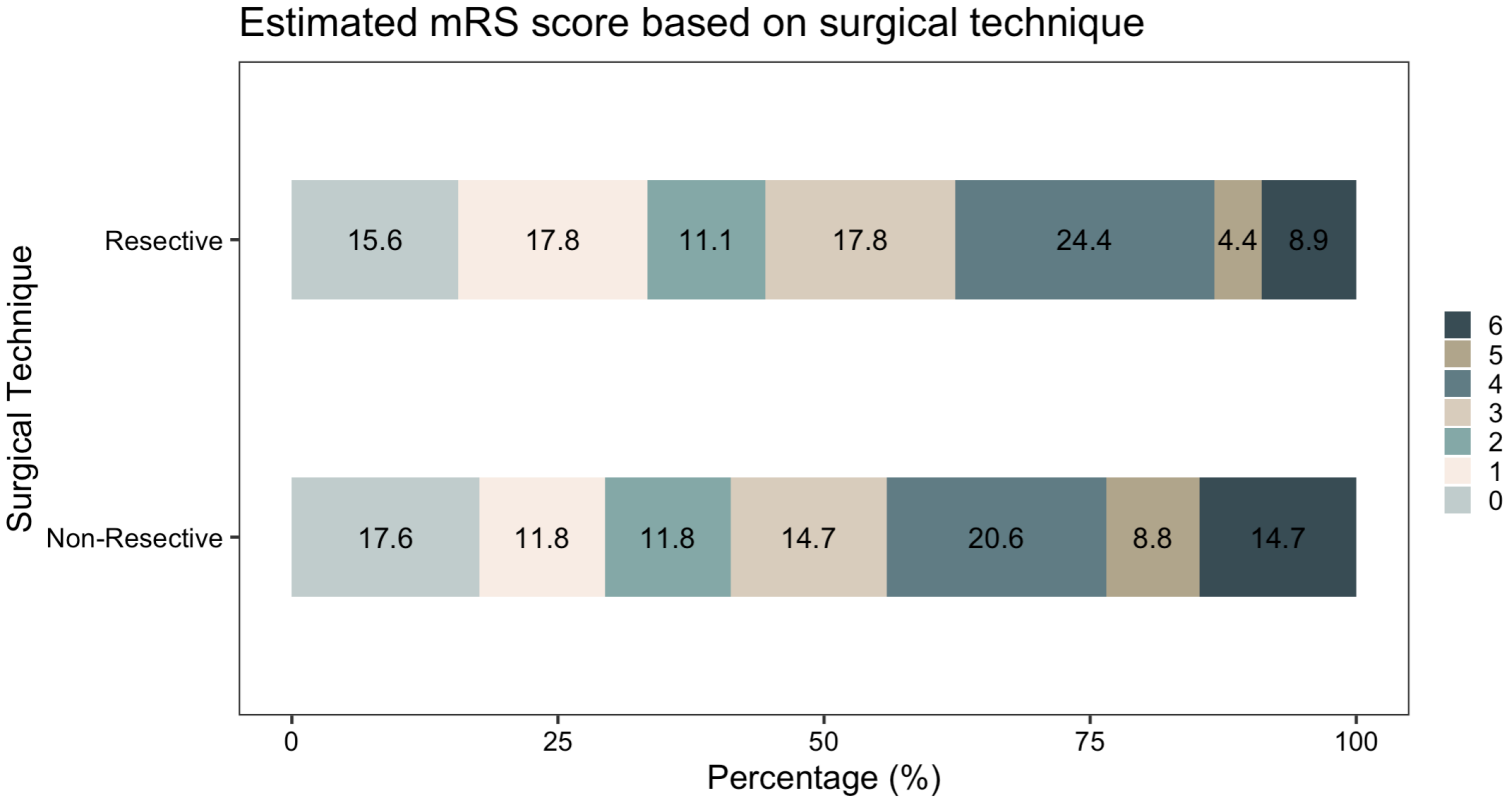
Comparison of mRS at last follow-up between resective and non-resective surgery.

Following initial intervention, SE was successfully aborted in 90.1%. Although resective techniques tended to have a higher likelihood of SE cessation, this was not statistically significant (93.9% vs. 83.9%, *p* = 0.071). Although MST achieved a markedly lower efficacy compared to other techniques, we did not identify a statistically significant difference between the overall rate of SE cessation after initial intervention between various surgical techniques. Among those who achieved SE cessation, time to SE cessation was shorter in the resective subgroup (Figure 4). Pooled proportions of SE cessation after initial intervention in studies with more than one participant was 95.2% [87.2%–99.7%] (Figure 5). Interstudy heterogeneity was low (I^2^ = 0%). There was no significant difference between resective and non-resective techniques on the test for subgroup difference (χ^2^ = 1.39, *p* = 0.24). In total, 20 patients (12.4%) required reoperation, of which 10 aimed to stop SE. Two underwent neuromodulation (DBS after failure of VNS or VNS after failure of corpus callosotomy). Seven underwent focal resection after failure of initial focal resection (3/7), MST (3/7) or SCS (1/7). Finally, one patient underwent hemispheric surgery after failure of focal resection to control SE. Mean time to reoperation was 12.5 ± 7.3 days. Reoperation successfully aborted SE in 8/10 patients. Resection was more likely to achieve seizure freedom postoperatively, compared with non-resective techniques (69.1% vs. 34.4%, *p* <0.0001). Functional outcomes were comparable between both cohorts (Figure 6). A favorable mRS was achieved in 44.3% of all patients. Patients with favorable mRS were less likely to be in SRSE, compared to those with poor outcomes (77.1% vs. 97.7%, *p* = 0.0088). At last follow-up, 14 (8.7%) patients had died, of which five were due to causes unrelated to epilepsy, six were due to postoperative persistent or recurrent SE, and three were ICU-related (i.e., ventilator-acquired pneumonia, tracheostomy-related bleed, and anoxic event due to mucus plug in tracheostomy tube). Patients who died of causes related to SE or epilepsy were significantly older than those who survived or died of unrelated causes (*p* = 0.0071).

### Prognostic factors

All patients with SE duration longer than one year were considered outliers and eliminated from our logistic regression models, resulting in the exclusion of six patients. Age at SE and time spent in SE prior to surgery were log-transformed to base two to normalize their distribution; therefore, the odds ratio (OR) reported for these variables corresponds to an increase by a factor of two. Older age at SE (OR = 1.384[1.046–1.832], *p* = 0.023) was an independent predictor of SE cessation, while new onset seizures (OR = 0.244[0.069–0.860], *p* = 0.028) was associated with a lower likelihood of SE cessation. Longer SE duration before surgery (OR = 0.603[0.362-1.003], *p* = 0.051) had a strong tendency to decrease the likelihood of SE cessation, but this did not reach significance. Resective surgery was not independently associated with SE cessation on multivariable analysis (Table 6). After exclusion of multimodal treatment, 149 patients were included in regression analysis for seizure freedom. Time in SE prior to surgery (OR = 0.597[0.416–0.856], *p* = 0.0060) and immediate SE cessation (OR = 3.736[1.323–10.548], *p* = 0.014) emerged as the only independent predictors of long-term seizure status on multivariable analysis (Table 6). Finally, our multivariable regression models on functional outcomes including 72 participants did not identify any independent predictors of favorable mRS at last follow-up (Table 6).

**Table 6:** Predictors of SE cessation after surgical intervention.

	Univariable model		Multivariable model	
	OR (95% CI)	*p*-value	OR (95% CI)	*p*-value
**Univariable and multivariable logistic regression for SE cessation**				
Age at SE†	1.176 (0.931–1.485)	0.17	1.384 (1.046–1.832)	0.023^*^
New-onset seizures	0.352 (0.117–1.054)	0.062	0.244 (0.069–0.860)	0.028^*^
Time in SE prior to surgery†	0.631 (0.413–0.966)	0.034^*^	0.603 (0.362–1.003)	0.051
Resective surgery	2.875 (0.958–8.630)	0.060	2.391 (0.703–8.129)	0.16
Diffuse slowing on EEG	0.394 (0.092–1.693)	0.21	-	-
**Univariable and multivariable logistic regression for seizure freedom (Engel I)**				
Resective surgery	3.908 (1.890–8.080)	0.0003^*^	-	-
Immediate effect	4.580 (2.007–10.453)	0.0005^*^	3.736 (1.323–10.548)	0.014^*^
Concordant MRI/EEG	3.502 (1.616–7.589)	0.0018^*^	2.312 (0.971–5.503)	0.058
Time in SE prior to surgery†	0.604 (0.436–0.838)	0.0032^*^	0.597 (0.416–0.856)	0.0060^*^
**Univariable and multivariable logistic regression for favorable mRS (0–2)**				
History of SE	0.321 (0.076–1.352)	0.12	0.375 (0.087–1.611)	0.18
Diffuse slowing on EEG	0.220 (0.050–0.961)	0.044	0.243 (0.055–1.075)	0.062

Univariable analyses of all covariates with *p* < 0.25 and multivariable analyses of all variables selected in more than five of the imputed datasets after forward stepwise selection are shown. SE, status epilepticus; EEG, electroencephalogram; MRI, magnetic resonance imaging. ^*^Statistically significant. ^†^The variable was log transformed to base 2.

## Discussion

In this systematic review and meta-analysis, we comprehensively analyzed the outcomes of patients who underwent emergency surgery while in RSE or SRSE. We demonstrated that initial surgical intervention successfully aborted SE in 90.1% of patients. New onset seizures leading to RSE/SRSE and younger age at SE were independently associated with a lower likelihood of SE cessation. Shorter duration of SE prior to surgery had a strong tendency to increase the likelihood of SE cessation but this did not reach statistical significance. Only duration of SE prior to surgery and immediate postoperative SE cessation were significantly associated with long-term seizure outcomes. In total, 44.3% of patients achieved a favorable mRS at last follow-up.

There is ongoing debate on indications and timing of surgery in patients with RSE and SRSE (10). In the absence of reliable prognostic factors, establishing the timepoint at which medical therapy (i.e., AED, IVAD) can be considered failure is challenging (23). Currently, surgery is considered only as last resort after weeks of RSE/SRSE (13). However, given the potential complications associated with prolonged seizures and aggressive ICU care (28, 29), some authors have proposed a shorter, two-week period after SRSE onset to consider surgical intervention (10, 30). We demonstrated that longer duration of SE prior to surgery tended to be associated with persistent SE and independently predicted worse long-term seizure outcomes, also suggesting an earlier consideration for epilepsy surgery. These findings suggest that within the presented cohort consisting mainly of patients who responded favorably to surgery, a shorter duration of SE was associated with improved outcomes. Furthermore, concordant findings on imaging and EEG also tended to increase the likelihood of long-term seizure control without reaching statistical significance. Overall, these findings suggest that for patients with known epilepsy and focal imaging and EEG findings, a low threshold for surgical resection should be adopted, as they may benefit from an early focal resection or hemispheric surgery not only for SE cessation, but also for long-term seizure freedom. Finally, contradictory to evidence from the literature, our analyses also revealed that older age was associated with improved SE outcomes. We hypothesize that a subset of pediatric patients with severe SRSE may have contributed to this finding. In fact, age at surgery did not emerge as a predictor of long-term seizure and functional outcomes, demonstrating that once SE is successfully aborted, age is not a predictor of long-term outcome.

Although open disconnective approaches and neuromodulation are usually reserved for more challenging cases, surgical approach (resective vs. non-resective) did not emerge as an independent predictor of outcome in any of our analyses and various surgical techniques demonstrated comparable efficacy in SE control. Thus, patients with unexplained new onset seizures such as new onset refractory status epilepticus (NORSE) with generalized ictal EEG findings and diffuse or normal imaging findings could still benefit from these approaches. However, our logistic regression models revealed that new-onset seizures were associated with worse outcomes; thus, clinical decision-making should still be made on a case-by-case basis given the lack of high-quality evidence guiding therapy in these patients, as well as the documented role of therapies such as the ketogenic diet and immunotherapy (31, 32). Some authors suggest the use of non-invasive neuromodulation techniques such as and transcranial magnetic stimulation (TMS) and electroconvulsive therapy (ECT) in the first week of SRSE, and consideration of neuromodulation techniques such as VNS, DBS and RNS after 2 weeks of therapy (33).

Although corpus callosotomy and MST remain viable options, their overall efficacy was equal or slightly inferior to neuromodulation alternatives that are thought to have a better safety profile. MST can be a useful adjunct to resective surgery when resection is limited by eloquent cortical areas. However, MST alone had markedly lower efficacy compared to the other techniques and led to reoperation with focal resection in 3/5 patients, all of which subsequently came out of SE. Corpus callosotomy is a palliative procedure with indications including certain epilepsy syndromes and certain seizure types (e.g., generalized atonic, tonic or tonic–clonic) that are not amenable to resection (34). In the context of RSE/SRSE with generalized seizures, corpus callosotomy may be considered to limit or reduce bilateral synchronous epileptic abnormalities or halt secondary seizure spread and potentially help with further lateralization and subsequent focal resection (35). Among the five patients that underwent initial corpus callosotomy, SE was successfully aborted in four, but only one patient underwent subsequent resection and achieved seizure freedom. One patient with persistent SE after corpus callosotomy underwent reoperation with VNS which successfully controlled SE. Considering the less invasive and less permanent nature of VNS and DBS, as well as their comparable efficacy to corpus callosotomy, they may be a more plausible option and considered earlier in the acute setting of RSE/SRSE, although further investigations in larger studies are required to draw firm conclusions.

The literature reporting on the efficacy of neuromodulation for RSE/SRSE is limited, and mechanisms by which neuromodulation affects SE remain to be elucidated (33). Despite limited evidence on its utility for RSE and SRSE, our findings suggest that neuromodulation is a viable alternative in patients who are not ideal candidates for open resective approaches with an overall SE cessation rate of 88.5% and an overall response rate of 74.2% in patients with prior epilepsy. The most commonly reported techniques in the literature are VNS and DBS. Current indications for VNS in epilepsy are absence of surgically remediable syndromes, bilateral and/or diffuse epileptic foci and generalized idiopathic epilepsy (36). In our study, patients who underwent VNS typically presented with generalized/multifocal seizures and diffuse or normal findings on EEG and neuroimaging. Thus, patients presenting with these clinical findings may benefit from an early consideration or implantation of VNS if other medical therapies fail. Alternatively, previous trials have demonstrated the safety and efficacy of DBS in epilepsy (16, 37). Similarly to VNS, CMN DBS can be a plausible option for patients with multifocal/generalized seizures or those that are not ideal candidates for surgery. Furthermore, a synergistic effect of DBS combined with other therapies such as anakinra has previously been documented (38). On the other hand, ANT DBS is a viable alternative to resection in patients with focal limbic epilepsy (e.g., temporal lobe epilepsy) that are not ideal candidates for resection. Interestingly, Stavropoulos et al. used ANT DBS in a patient with focal epilepsy due to FCD in the acute setting of SRSE to abort SE and perform further workup for subsequent resection of the lesion (39), suggesting the use of neuromodulation in conjunction with surgical resection for the management of these patients. In addition to thalamic targets, two patients with EPC received unilateral DBS of GPi and CZi which are unconventional targets for epilepsy and are commonly used for movement disorders (40–45). Seizures were permanently stopped in one patient and reduced in the other, suggesting DBS of motor targets as a potential alternative in patients with EPC or focal motor seizures originating from the sensorimotor cortex, although further investigations are required to assess the efficacy of these targets.

Chronic subdural cortical stimulation (SCS) is an open loop system that can be used in patients with epileptic foci within eloquent cortical areas who are not ideal candidates for resective surgery (46, 47). Alternatively, RNS is a closed-loop system that delivers stimulation to cortical or subcortical epileptic focus or foci that cannot be surgically resected upon detection of seizure activity (17). Only a total of 10 cases (four cases with RNS and six with SCS) undergoing these techniques for RSE/SRSE have been published in the literature. Both techniques were effective in controlling SE, with only one patient with severe disease and multifocal seizures experiencing persistent SRSE following SCS. In terms of long-term seizure control, SCS performed slightly better than RNS, although the limited number of patients in each subgroup precluded more robust comparisons. Compared to SCS, RNS offers the possibility to stimulate both cortical and deep subcortical areas using cortical strip leads and depth leads, respectively. It may also be better tolerated by avoiding the commonly known side effects of chronic stimulation. However, the comparison of chronic and responsive neurostimulation is a topic of debate and remains to be discussed in comparative trials with a larger number of patients (46).

Given the lack of consensus regarding the use of neuromodulation techniques for RSE and SRSE, stimulation parameters and titration periods were highly variable across studies, and we did not identify any association between different strategies and final outcomes. However, an aggressive approach may be prioritized to prevent or minimize the risks of prolonged RSE/SRSE unless if complications occur.

The literature on the surgical treatment of RSE/SRSE consists mainly of case reports with high risk of publication bias. For instance, most case reports present favorable outcomes while case series are more likely to include patients that did not respond favorably to surgical treatment (48, 49). Thus, we hypothesize that the cohort included in our study may represent a subset of patients that respond more favorably to surgical treatment. On the other hand, since surgery is often considered as last resort for patients with severe disease, included participants may represent a subset of patients with more severe SE that is more difficult to manage medically. For instance, we observed stark differences in seizure semiology and etiology of patients included in this study and a recently published IPDMA on the outcome of patients with SRSE in which most included patients underwent medical treatment (23). Thus, findings from our study should be interpreted with caution as they may be more applicable to patients with severe disease that are inherently better candidates for epilepsy surgery. Given the rarity of RSE and SRSE and the high variability between individual patients, prospective and randomized controlled trials are challenging, particularly for the surgical management of these conditions. Thus, despite some limitations, a meta-analysis gathering IPD on the case reports and small cohorts provides considerable value in the assessment of the safety and efficacy of surgery for RSE/SRSE. In this regard, our study helped characterize a subset of patients that respond more favorably to epilepsy surgery and identify prognostic factors, which could potentially help with the establishment of more specific indications for surgery.

### Limitations

Our study had several limitations. Mainly, the current literature on the surgical treatment of RSE and SRSE is limited exclusively to single-center, retrospective studies, with major risk of publication bias significantly favoring surgical treatment. Thus, it is difficult to make strong recommendations on the management of these patients based on currently available evidence. Additionally, several neuromodulation techniques are not available in various centers across the world and clinical decision-making is often made on a case-by-case basis based on available techniques and personal preferences. Furthermore, data on long-term functional and cognitive outcomes are scarce. Almost all mRS scores were mostly estimated based on data presented in each study, potentially leading to some inaccuracy in our findings. Our regression models for functional outcomes included less than half of all participants and were certainly underpowered to identify viable predictors of outcome. Furthermore, neuropsychological outcomes were scarcely reported. Thus, we were unable to collect and summarize this data and perform analyses on associations between various medical and surgical treatments and long-term cognitive outcomes. Further large-scale, prospective trials are required to better characterize the cognitive trajectories in patients with RSE/SRSE. Finally, given the limited data on AED and IVAD use, we were unable to study associations between types and dosages of medical therapies and long-term patient outcomes. This might be of particular interest for neuromodulation where the distinction between the therapeutic benefit of neuromodulation and medical therapy is often difficult to make.

## Conclusion

We performed the first IPDMA comparing all neurosurgical techniques used for the acute treatment of RSE and SRSE. The existing data suggest that surgery can safely and effectively control SE and achieve long-term seizure control in patients with RSE and SRS. We identified no significant difference in SE cessation rates between resective and non-resective techniques, but open resection achieved better long-term seizure outcomes. Resection should be considered early on in patients presenting with focal seizures and concordant semiology, imaging, and EEG. Neuromodulation and other disconnective techniques can be considered in patients with new-onset SE and normal/non-focal findings on imaging and EEG or those with epileptic foci located in eloquent cortex. Further investigation of the utility of these techniques in larger, prospective trials is warranted to provide a robust evaluation of the efficacy and timing of surgical techniques in SE control and the long-term seizure, functional and cognitive outcome of these patients.

## Data availability statement

The original contributions presented in the study are included in the article/Supplementary material, further inquiries can be directed to the corresponding author.

## Author contributions

FN: Formal analysis, Methodology, Writing – original draft, Writing – review & editing. AlH: Data curation, Writing – review & editing. LS: Data curation, Writing – review & editing. NS: Data curation, Writing – review & editing. CK: Writing – review & editing. TH: Data curation, Writing – review & editing. AK: Writing – review & editing. GP-T: Writing – review & editing. LD: Writing – review & editing. PM: Writing – review & editing. DN: Writing – review & editing. GI: Writing – review & editing. AF: Methodology, Supervision, Writing – review & editing. ArH: Methodology, Supervision, Writing – review & editing. AW: Methodology, Supervision, Writing – review & editing. AB: Methodology, Supervision, Writing – review & editing.
