# Supplementary material for: Outcome of emergency neurosurgery in patients with refractory and super-refractory status epilepticus: a systematic review and individual participant data meta-analysis
Source: Front Neurol. 2024 May 28;15:1403266. doi: 10.3389/fneur.2024.1403266 (PMC11165020; doi:10.3389/fneur.2024.1403266)
Supplement: Supplementary file 1 [file Data_Sheet_1.docx]

Table S1. Search strategy and keywords included.

| Database | **Search Term** |
| --- | --- |
| Medline | \| 1 \| Deep Brain Stimulation/ or Vagus Nerve Stimulation/ or nerve stimulation/ or functional electrical stimulation/ or nerve cell stimulation/ or exp electric stimulation/ or implantable neurostimulators/ or electric stimulation therapy/ or transcranial direct current stimulation/ or transcutaneous electric nerve stimulation/ \| \| --- \| --- \| \| 2 \| vagal activit*.tw,kf. \| \| 3 \| neuromodulat*.tw,kf. \| \| 4 \| ((brain or nerve or neural or vagal or vagus or electric*) adj2 (stimul* or excit*)).tw,kf. \| \| 5 \| (VNS or DBS).tw,kf. \| \| 6 \| neurostimulat*.tw,kf. \| \| 7 \| or/1-6 [neuromodulation] \| \| 8 \| ((Resective or epilep* or neurologic* or hemispheric or temporal or lobe) adj2 (surgery or disconnection or ablation or removal or resect*)).tw,kf. \| \| 9 \| neurosurg*.tw,kf. \| \| 10 \| (hemispherectom* or hemispherotom* or lobectom*).tw,kf. \| \| 11 \| multiple subpial transections.tw,kf. \| \| 12 \| acute surgical resection.tw,kf. \| \| 13 \| Neurosurgical Procedures/ or Hemispherectomy/ or Anterior Temporal Lobectomy/ \| \| 14 \| or/8-13 [neurosurgery] \| \| 15 \| 7 or 14 \| \| 16 \| ("15699401" or "18585939" or "33955717" or "31403465" or "26543817" or "28357637").ui. \| \| 17 \| 15 and 16 \| \| 18 \| exp status epilepticus/ \| \| 19 \| (status adj epileptic*).tw,kf. \| \| 20 \| ((continu* or prolong* or incontrol* or uncontrol*) adj2 seizure*).tw,kf. \| \| 21 \| epilepsia partialis continua.tw,kf. \| \| 22 \| or/18-21 [Status epilepticus] \| \| 23 \| 15 and 22 \| |
| Embase | \| 1 \| brain depth stimulation/ \| \| --- \| --- \| \| 2 \| vagus nerve stimulation/ \| \| 3 \| nerve stimulation/ \| \| 4 \| functional electrical stimulation/ \| \| 5 \| nerve cell stimulation/ \| \| 6 \| electrostimulation/ \| \| 7 \| implantable neurostimulator/ \| \| 8 \| electrotherapy/ \| \| 9 \| transcranial direct current stimulation/ \| \| 10 \| transcutaneous electrical nerve stimulation/ \| \| 11 \| vagal activit*.tw,kf. \| \| 12 \| neuromodulat*.tw,kf. \| \| 13 \| ((brain or nerve or neural or vagal or vagus or electric*) adj2 (stimul* or excit*)).tw,kf. \| \| 14 \| (VNS or DBS).tw,kf. \| \| 15 \| neurostimulat*.tw,kf. \| \| 16 \| or/1-15 \| \| 17 \| ((Resective or epilep* or neurologic* or hemispheric or temporal or lobe) adj2 (surgery or disconnection or ablation or removal or resect*)).tw,kf. \| \| 18 \| neurosurg*.tw,kf. \| \| 19 \| (hemispherectom* or hemispherotom* or lobectom*).tw,kf. \| \| 20 \| multiple subpial transections.tw,kf. \| \| 21 \| acute surgical resection.tw,kf. \| \| 22 \| neurosurgery/ \| \| 23 \| hemispherectomy/ \| \| 24 \| temporal lobectomy/ \| \| 25 \| or/17-24 \| \| 26 \| 16 or 25 \| \| 27 \| epileptic state/ \| \| 28 \| (status adj epileptic*).tw,kf. \| \| 29 \| ((continu* or prolong* or incontrol* or uncontrol*) adj2 seizure*).tw,kf. \| \| 30 \| epilepsia partialis continua.tw,kf. \| \| 31 \| ESES.tw,kf. \| \| 32 \| or/27-31 \| \| 33 \| 26 and 32 \| |
| Cochrane SR | \| 1 \| Deep Brain Stimulation.kw,tw. \| \| --- \| --- \| \| 2 \| Vagus Nerve Stimulation.tw,kw. \| \| 3 \| nerve stimulation.tw,kw. \| \| 4 \| functional electrical stimulation.tw,kw. \| \| 5 \| nerve cell stimulation.tw,kw. \| \| 6 \| electric stimulation.tw,kw. \| \| 7 \| Electroshock.tw,kw. \| \| 8 \| Chronaxy.tw,kw. \| \| 9 \| implantable neurostimulators.tw,kw. \| \| 10 \| electric stimulation therapy.tw,kw. \| \| 11 \| transcranial direct current stimulation.tw,kw. \| \| 12 \| transcutaneous electric nerve stimulation.tw,kw. \| \| 13 \| vagal activit*.tw. \| \| 14 \| neuromodulat*.tw. \| \| 15 \| ((brain or nerve or neural or vagal or vagus or electric*) adj2 (stimul* or excit*)).tw. \| \| 16 \| (VNS or DBS).tw. \| \| 17 \| neurostimulat*.tw. \| \| 18 \| 1 or 2 or 3 or 4 or 5 or 6 or 7 or 8 or 9 or 10 or 11 or 12 or 13 or 14 or 15 or 16 or 17 \| \| 19 \| ((Resective or epilep* or neurologic* or hemispheric or temporal or lobe) adj2 (surgery or disconnection or ablation or removal or resect*)).tw. \| \| 20 \| neurosurg*.tw. \| \| 21 \| (hemispherectom* or hemispherotom* or lobectom*).tw. \| \| 22 \| multiple subpial transections.tw. \| \| 23 \| acute surgical resection.tw. \| \| 24 \| ("Neurosurgical Procedures" or Hemispherectomy or "Anterior Temporal Lobectomy").kw,tw. \| \| 25 \| or/19-24 [neurosurgery] \| \| 26 \| ("Status Epilepticus" or "Epilepsia Partialis Continua").tw,kw. \| \| 27 \| (status adj epileptic*).tw. \| \| 28 \| ((continu* or prolong* or incontrol* or uncontrol*) adj2 seizure*).tw. \| \| 29 \| epilepsia partialis continua.tw. \| \| 30 \| ESES.tw. \| \| 31 \| or/26-30 [Status epilepticus] \| \| 32 \| 18 or 25 \| \| 33 \| 31 and 32 \| |
| Web of Science | \| 1 \| TS=((Resective OR epilep* OR neurologic* OR hemispheric OR tempORal OR lobe) NEAR2 (surgery OR disconnection OR ablation OR removal OR resect*)) \| \| --- \| --- \| \| 2 \| TS=neurosurg* \| \| 3 \| TS=(hemispherectom* OR hemispherotom* OR lobectom*) \| \| 4 \| TS="multiple subpial transections" \| \| 5 \| TS="acute surgical resection" \| \| 6 \| #1 OR #2 OR #3 OR #4 OR #5 \| \| 7 \| TS=neurostimulat* \| \| 8 \| TS=(VNS OR DBS) \| \| 9 \| TS=((brain OR nerve OR neural OR vagal OR vagus OR electric*) NEAR2 (stimul* OR excit*)) \| \| 10 \| TS=neuromodulat* \| \| 11 \| TS="vagal activit*" \| \| 12 \| TS=("Deep Brain Stimulation" OR "Vagus Nerve Stimulation" OR "nerve stimulation" OR "functional electrical stimulation" OR "nerve cell stimulation" OR "electric stimulation" OR "implantable neurostimulatORs" OR "electric stimulation therapy" OR "transcranial direct current stimulation" OR "transcutaneous electric nerve stimulation") \| \| 13 \| #7 OR #8 OR #9 OR #10 OR #11 OR #12 \| \| 14 \| #6 OR #13 \| \| 15 \| TS=(status NEAR epileptic*) \| \| 16 \| TS=((continu* OR prolong* OR incontrol* OR uncontrol*) NEAR2 seizure*) \| \| 17 \| #15 OR #16 \| \| 18 \| #14 AND #17 \| |

Table S2. Characteristics and Outcomes of all included studies

| **Study ID** | **Country** | **No. of Participants** | **Age (years)** | **SRSE (%)** | **SE Classification** | **Surgical Intervention** | **SE Cessation (%)** | **Overall Seizure Freedom (%)** | **Favorable Outcome (mRS 0-2) (%)** |
| --- | --- | --- | --- | --- | --- | --- | --- | --- | --- |
| Mamaril-Davis et al. 2022 | USA | 1 | 20.0 | 100.0% | FTBTC SE (n=1) | RNS (n=1) | 100.0% | 0.0% | 100.0% |
| Alexopoulos et al. 2005 | USA | 10 | 6.9 ± 7.1 | 100.0% | FIAS SE (n=2), focal motor SE (n=5), FTBTC SE (n=3) | Focal resection (n=4), hemispherectomy (n=6) | 100.0% | 70.0% | NA |
| Alsaadi et al. 2015 | UAE | 1 | 46.0 | 100.0% | Generalized NCSE (n=1) | VNS (n=1) | 100.0% | 100.0% | NA |
| Atkinson et al. 2012 | USA | 1 | 20.0 | 100.0% | FTBTC SE (n=1) | Focal resection (n=1) | 100.0% | 0.0% | 100.0% |
| Barros et al. 2014 | Portugal | 1 | 7.0 | 100.0% | FTBTC SE (n=1) | Focal resection (n=1) | 100.0% | 0.0% | 0.0% |
| Bhave et al. 2023 | USA | 1 | 52.0 | 100.0% | FIAS SE with motor symptoms (n=1) | Focal resection (n=1) | 100.0% | 100.0% | 0.0% |
| Bick et al. 2016 | USA | 1 | 60.0 | 100.0% | FIAS SE (n=1) | Focal resection (n=1) | 100.0% | 100.0% | 100.0% |
| Botre et al. 2017 | India | 2 | 4.0 ± 4.2 | 100.0% (n=1), NA (n=1) | EPC (n=1), FTBTC SE (n=1) | Hemispherectomy (n=2) | 100.0% | 100.0% | 0.0% (n=1), NA (n=1) |
| Chandra et al. 2011 | India | 1 | 0.1 | 100.0% | FTBTC SE (n=1) | Focal resection (n=1) | 100.0% | 100.0% | 0.0% |
| Lee et al. 2016 | Taiwan | 1 | 17.0 | 100.0% | Generalized NCSE (n=1) | DBS (n=1) | 100.0% | 0.0% | NA |
| Hect et al. 2022 | USA | 1 | 11.0 | 100.0% | Generalized NCSE (n=1) | VNS (n=1) | 0.0% | 0.0% | NA |
| Ernst et al. 2019 | USA | 1 | 37.0 | 100.0% | EPC (n=1) | RNS (n=1) | 100.0% | 0.0% | 100.0% |
| Mohamed et al. 2007 | Canada | 1 | 10.0 | 100.0% | FTBTC SE (n=1) | Focal resection (n=1) | 100.0% | 0.0% | 100.0% |
| Yuan et al. 2019 | China | 1 | 25.0 | 100.0% | Generalized convulsive SE (n=1) | DBS (n=1) | 100.0% | 0.0% | 0.0% |
| Stavropoulos et al. 2019 | UK | 1 | 15.0 | 100.0% | FTBTC SE (n=1) | DBS (n=1) | 100.0% | 0.0% | NA |
| Shrader et al. 2009 | Canada | 3 | 3.4 ± 2.3 | 100.0% | FIAS SE (n=2), FTBTC SE (n=1) | Focal resection (n=2), MST (n=1) | 33.3% | 33.3% | 33.3% |
| Larijani et al. 2019 | Iran | 1 | 16.0 | 100.0% | Generalized convulsive SE (n=1) | Corpus callostomy (n=1) | 100.0% | 0.0% | 0.0% |
| Nayak et al. 2017 | India | 1 | 56.0 | 100.0% | FTBTC SE (n=1) | Hemispherectomy (n=1) | 100.0% | 100.0% | 0.0% |
| Kurukumbi et al. 2019 | USA | 1 | 25.0 | 100.0% | Generalized convulsive SE (n=1) | VNS (n=1) | 100.0% | 0.0% | 0.0% |
| Weimer et al. 2008 | USA | 1 | 45.0 | 100.0% | FIAS SE (n=1) | Focal resection (n=1) | 100.0% | 100.0% | 100.0% |
| Imbach et al. 2019 | Switzerland | 1 | 66.0 | 100.0% | Generalized NCSE (n=1) | DBS (n=1) | 100.0% | 100.0% | NA |
| Yonamoto et al. 2022 | Japan | 1 | 58.0 | 100.0% | FIAS SE (n=1) | Focal resection (n=1) | 100.0% | 100.0% | NA |
| Greiner et al. 2012 | USA | 1 | 9.0 | 100.0% | Generalized NCSE (n=1) | Corpus callostomy (n=1) | 100.0% | 0.0% | 100.0% |
| Nahab et al. 2008 | USA | 1 | 57.0 | 100.0% | FIAS SE with motor symptoms (n=1) | Focal resection (n=1) | 100.0% | 100.0% | 0.0% |
| Lehtimaki et al. 2016 | Finland | 1 | 17.0 | 100.0% | Generalized convulsive SE (n=1) | DBS (n=1) | 100.0% | 0.0% | NA |
| Franzini et al. 2008 | Italy | 1 | 22.0 | 0.0% | EPC (n=1) | DBS (n=1) | 100.0% | 0.0% | NA |
| Valentin et al. 2012 | UK | 1 | 27.0 | 100.0% | Generalized myoclonic SE (n=1) | DBS (n=1) | 100.0% | 100.0% | 0.0% |
| Sa et al. 2019 | UK | 2 | 7.0 ± 2.8 | 100.0% | FTBTC SE (n=1), general convulsive SE (n=1) | DBS (n=2) | 50.0% | 0.0% | 50.0% |
| Specchio et al. 2020 | Italy | 2 | 8.3 ± 11.0 | 100.0% | FTBTC SE (n=1), generalized myoclonic SE (n=1) | VNS (n=2) | 100.0% | 0.0% | NA |
| Al-Attas et al. 2022 | Saudi Arabia | 1 | 16.0 | 100.0% | Generalized convulsive SE (n=1) | VNS (n=1) | 100.0% | 100.0% | NA |
| De Herdt et al. 2009 | Belgium | 1 | 7.0 | 100.0% | Generalized NCSE (n=1) | VNS (n=1) | 100.0% | 100.0% | NA |
| Yamazoe et al. 2017 | Japan | 1 | 24.0 | 100.0% | FTBTC SE (n=1) | Corpus callostomy (n=1) | 0.0% | 0.0% | NA |
| Mostacci et al. 2019 | Italy | 1 | 16.0 | 100.0% | Generalized convulsive SE (n=1) | VNS (n=1) | 100.0% | 0.0% | 0.0% |
| Luo et al. 2022 | China | 1 | 2.4 | 100.0% | Generalized convulsive SE (n=1) | VNS (n=1) | 100.0% | 100.0% | NA |
| Yazdi et al. 2016 | USA | 1 | 67.0 | 100.0% | FIAS SE with motor symptoms (n=1) | VNS (n=1) | 100.0% | 100.0% | 100.0% |
| Espino et al. 2022 | Canada | 1 | 37.0 | 100.0% | FTBTC SE (n=1) | VNS (n=1) | 100.0% | 0.0% | NA |
| Braakman et al. 2018 | Netherlands | 1 | 16.0 | 100.0% | Generalized NCSE (n=1) | VNS (n=1) | 100.0% | 100.0% | 0.0% |
| Dhaliwal et al. 2020 | USA | 1 | 30.0 | 100.0% | Generalized NCSE (n=1) | VNS (n=1) | 100.0% | 100.0% | 100.0% |
| Basha et al. 2017 | USA | 9 | 51.3 ± 16.2 | 100.0% | FIAS SE (n=4), FTBTC SE (n=5) | Focal resection (n=9) | 88.9% | 55.6% | 33.3% |
| Bhatia et al. 2013 | USA | 15 | 9.5 ± 5.5 | 100.0% | EPC (n=3), FIAS SE (n=7), FTBTC SE (n=5) | Focal resection (n=12), hemispherectomy (n=1), MST (n=2) | 86.7% | 53.3% | NA |
| Chang et al. 2019 | China | 3 | 24.0 ± 2.6 | NA | EPC (n=2), FIAS SE (n=1) | Subdural cortical stimulation (n=3) | 100.0% | 33.3% | NA |
| Cheong et al. 2009 | Australia | 1 | 27.0 | 0.0% | EPC (n=1) | Hemispherectomy (n=1) | 100.0% | 100.0% | NA |
| Costello et al. 2006 | USA | 1 | 36.0 | 100.0% | FIAS SE with motor symptoms (n=1) | MST + (n=1) | 100.0% | 100.0% | 100.0% |
| Cuddapah et al. 2015 | USA | 1 | 0.1 | 100.0% | FIAS SE (n=1) | Hemispherectomy (n=1) | 100.0% | 100.0% | 100.0% |
| Cuello-Oderiz et al. 2015 | Argentina | 3 | 15.7 ± 6.8 | 100.0% | FIAS SE with motor symptoms (n=2), focal motor SE (n=1) | Focal resection (n=1), hemispherectomy (n=2) | 100.0% | 66.7% | NA |
| D'Giano et al. 2001 | Argentina | 1 | 6.0 | 100.0% | EPC (n=1) | MST + (n=1) | 100.0% | 100.0% | NA |
| Duane et al. 2004 | USA | 1 | 7.0 | 100.0% | FIAS SE (n=1) | Hemispherectomy (n=1) | 100.0% | 100.0% | 0.0% |
| Gorman et al. 1992 | USA | 1 | 9.8 | 100.0% | FIAS SE with motor symptoms (n=1) | Focal resection (n=1) | 100.0% | 100.0% | 0.0% |
| Graley et al. 2021 | USA | 1 | 26.0 | 100.0% | Focal motor SE (n=1) | RNS (n=1) | 100.0% | 0.0% | 0.0% |
| Grubor et al. 2022 | Sweden | 1 | 24.0 | 100.0% | FIAS SE with motor symptoms (n=1) | Focal resection (n=1) | 100.0% | 100.0% | 100.0% |
| Guvenc et al. 2020 | Turkey | 1 | 27.0 | 100.0% | Focal motor SE (n=1) | Focal resection (n=1) | 100.0% | 0.0% | NA |
| Jagtap et al. 2021 | India | 10 | 5.9 ± 4.6 | 60.0% | FIAS SE (n=2), focal motor SE (n=8) | Focal resection (n=3), hemispherectomy (n=7) | 100.0% | 90.0% | NA |
| Juhasz et al. 2013 | USA | 1 | 56.0 | 0.0% | FIAS SE (n=1) | Focal resection (n=1) | 100.0% | 100.0% | 100.0% |
| Krsek et al. 2002 | Czech Republic | 1 | 0.3 | 100.0% | EPC (n=1) | Focal resection (n=1) | 100.0% | 100.0% | 0.0% |
| Lega et al. 2009 | USA | 2 | 43.0 (n=1) | 0.0% | EPC (n=2) | Focal resection (n=2) | 100.0% | 0.0% | 100.0% |
| Legatt et al. 1996 | USA | 1 | 7.0 | 0.0% | EPC (n=1) | Focal resection (n=1) | 100.0% | 100.0% | 100.0% |
| Lupashko et al. 2011 | USA | 1 | 5.0 | 100.0% | EPC (n=1) | Hemispherectomy (n=1) | 100.0% | 100.0% | 0.0% |
| Ma et al. 2001 | USA | 3 | 26.0 ± 4.6 | 100.0% | FIAS SE with motor symptoms (n=1), FTBTC SE (n=1), generalized convulsive SE (n=1) | Corpus callostomy (n=2), MST + (n=1) | 100.0% | 66.7% | NA |
| Mcginity et al. 2016 | USA | 2 | 43.0 ± 7.1 | 100.0% | EPC (n=1), FTBTC SE (n=1) | Focal resection (n=1), hemispherectomy (n=1) | 100.0% | 100.0% | 50.0% |
| Molyneux et al. 1998 | UK | 1 | 19.0 | 100.0% | EPC (n=1) | MST (n=1) | 100.0% | 100.0% | 0.0% |
| Mohamed et al. 2007 | Canada | 4 | 7.0 ± 5.6 | 100.0% | EPC (n=1), FIAS SE (n=1), FTBTC SE (n=2) | Focal resection (n=4) | 75.0% | 100.0% (n=3), NA (n=1) | NA |
| Ng et al. 2006 | USA | 3 | 3.8 ± 3.3 | 66.7% | EPC (n=1), FIAS SE (n=2) | Focal resection (n=1), hemispherectomy (n=1), MST (n=1) | 66.7% | 66.7% | 100.0% (n=2), NA (n=1) |
| Ng et al. 2007 | USA | 1 | 48.0 | 0.0% | EPC (n=1) | MST + (n=1) | 100.0% | 100.0% | NA |
| O'Neill et al. 2011 | USA | 1 | 23.0 | 100.0% | Generalized convulsive SE (n=1) | VNS (n=1) | 100.0% | 0.0% | 100.0% |
| Patwardhan et al. 2005 | USA | 1 | 30.0 | 100.0% | Generalized convulsive SE (n=1) | VNS (n=1) | 100.0% | 100.0% | 0.0% |
| Peterson et al. 2019 | USA | 1 | 0.0 | 100.0% | FTBTC SE (n=1) | Focal resection (n=1) | 100.0% | 100.0% | 100.0% |
| San-Juan et al. 2019 | USA | 1 | 20.0 | 100.0% | FIAS SE (n=1) | Focal resection (n=1) | 100.0% | 100.0% | 100.0% |
| Suchdev et al. 2021 | USA | 3 | 35.7 ± 18.4 | 100.0% | FIAS SE (n=1), FTBTC SE (n=2) | Focal resection (n=3) | 100.0% | 66.7% | 33.3% |
| Szabo et al. 2019 | USA | 1 | 23.0 | 100.0% | EPC (n=1) | Hemispherectomy (n=1) | 100.0% | 100.0% | 0.0% |
| Timer et al. 2018 | Turkey | 1 | 9.0 | 100.0% | FIAS SE (n=1) | Focal resection (n=1) | 100.0% | 100.0% | NA |
| Uysal et al. 2017 | USA | 1 | 61.0 | 100.0% | FIAS SE (n=1) | Focal resection (n=1) | 100.0% | NA | NA |
| Winston et al. 2000 | USA | 1 | 13.0 | 100.0% | Generalized convulsive SE (n=1) | VNS (n=1) | 100.0% | 0.0% | NA |
| Yang et al. 2021 | USA | 1 | 22.0 | 100.0% | EPC (n=1) | RNS (n=1) | 100.0% | 0.0% | NA |
| Schrader et al. 2006 | USA | 1 | 26.0 | 100.0% | Generalized convulsive SE (n=1) | Subdural cortical stimulation (n=1) | 0.0% | 0.0% | 0.0% |
| Valentin et al. 2015 | UK | 2 | 20.5 ± 0.7 | 100.0% | EPC (n=2) | Subdural cortical stimulation (n=2) | 100.0% | 0.0% | 100.0% |
| Courtin et al. 2022 | France | 1 | 32.0 | 0.0% | EPC (n=1) | DBS (n=1) | 0.0% | 0.0% | NA |
| Desbiens et al. 1993 | Australia, USA, Canada | 4 | 11.0 ± 7.7 | 100.0% (n=1), NA (n=3) | EPC (n=1), focal motor SE (n=3) | Focal resection (n=4) | 75.0% | 50.0% | 0.0% (n=1), NA (n=3) |
| Stone et al. 1986 | USA | 1 | 24.0 | 0.0% | FTBTC SE (n=1) | Focal resection (n=1) | 100.0% | 100.0% | 100.0% |
| Zamponi et al. 2008 | Italy | 2 | 0.7 ± 0.1 | 100.0% | Generalized convulsive SE (n=2) | VNS (n=2) | 100.0% | 0.0% | NA |
| De Bendictis et al. 2013 | Italy | 4 | 6.0 ± 6.4 | 0.0% | EPC (n=4) | VNS (n=4) | 100.0% | 25.0% | 66.7% (n=3), NA (n=1) |
| Cherian et al. 2009 | USA | 1 | 0.7 | 100.0% | FTBTC SE (n=1) | Focal resection (n=1) | 0.0% | 0.0% | 0.0% |
| Marashly et al. 2017 | USA | 1 | 3.0 | 100.0% | Focal motor SE (n=1) | Focal resection (n=1) | 100.0% | 100.0% | NA |
| Samanta et al. 2018 | USA | 1 | 13.0 | 100.0% | Focal motor SE (n=1) | Focal resection (n=1) | 100.0% | 0.0% | 0.0% |
| Rodriguez-Villar et al. 2023 | UK | 1 | 54.0 | 100.0% | Generalized convulsive SE (n=1) | VNS (n=1) | 100.0% | NA | NA |
| Jindal et al. 2023 | UK | 1 | 30.0 | 100.0% | Generalized myoclonic SE (n=1) | VNS (n=1) | 100.0% | 100.0% | 0.0% |
| Mehboob et al. 2023 | UK | 1 | 54.0 | 100.0% | Generalized convulsive SE (n=1) | VNS (n=1) | 100.0% | 0.0% | 100.0% |
| Melinda Furlanis et al. 2023 | Italy | 7 | 4.4 ± 4.8 | 100.0% | FTBTC SE (n=1), focal motor SE (n=3), generalized myoclonic SE (n=1), unknown (n=2) | VNS (n=7) | 71.4% | 28.6% | 0.0% (n=4), NA (n=3) |

Table S3. Clinical characteristics of patients stratified by their SE outcome following initial intervention.

|  | **SE cessation after initial surgery** | | | | |
| --- | --- | --- | --- | --- | --- |
| **Characteristic** | **Yes**  **N = 145** | **N** | **No**  **N = 16** | **N** | **p value** |
| **Sex (F)** | 55 (46.6%) | 118 | 6 (46.2%) | 13 | >0.99 |
| **Age at SE onset (years)** |  | 144 |  | 16 | 0.14 |
| Median (IQR) | 15.5 (6.0-26.3) |  | 5.5 (2.5-21.8) |  |  |
| Mean (SD) | 19.8 (18.4) |  | 13.1 (15.4) |  |  |
| Range | 0.0-68.0 |  | 0.6-57.0 |  |  |
| **New-onset seizures** | 34 (23.4% | 145 | 8 (50.0%) | 16 | 0.033* |
| **History of SE** | 20 (18.3%) | 109 | 1 (8.3%) | 12 | 0.69 |
| **SRSE** | 121 (87.7%) | 138 | 15 (93.8%) | 16 | 0.76 |
| **Time in SE prior to surgery (days)** |  | 118 |  | 14 | 0.050* |
| Median (IQR) | 21.0 (12.0-42.0) |  | 37.5 (20.0-90.0) |  |  |
| Mean (SD) | 113.7 (418.6) |  | 84.4 (111.4) |  |  |
| Range | 2.0-3468.0 |  | 14.0-420.0 |  |  |
| **SE semiology classification** |  | 144 |  | 15 | 0.51 |
| FTBTC SE | 30 (20.8%) |  | 6 (40.0%) |  |  |
| FIAS SE | 38 (26.4%) |  | 2 (13.3%) |  |  |
| Focal aware SE | 50 (34.7%) |  | 4 (26.7%) |  |  |
| Generalized NCSE | 7 (4.9%) |  | 1 (6.7%) |  |  |
| Generalized myoclonic SE | 4 (2.8%) |  | 0 (0.0%) |  |  |
| Generalized convulsive SE | 15 (10.4%) |  | 2 (13.3%) |  |  |
| **EEG Localization** |  | 144 |  | 15 | >0.99 |
| Focal | 115 (79.9%) |  | 12 (80.0%) |  |  |
| Generalized | 29 (20.1%) |  | 3 (20.0%) |  |  |
| **Focal slowing** | 14 (11.5%) | 122 | 0 (0.0%) | 10 | 0.60 |
| **Diffuse slowing** | 17 (13.9%) | 122 | 2 (20.0%) | 10 | 0.64 |
| **MRI finding** |  | 122 |  | 13 | 0.040* |
| Focal | 71 (58.2%) |  | 4 (30.8%) |  |  |
| Diffuse | 31 (25.4%) |  | 3 (23.1%) |  |  |
| Negative | 20 (16.4%) |  | 6 (46.2%) |  |  |
| **Concordant MRI-EEG findings** | 54 (48.2%) | 112 | 3 (25.0%) | 12 | 0.22 |
| **Etiology** |  | 138 |  | 15 | 0.15 |
| Known epilepsy | 74 (53.6%) |  | 6 (40.0%) |  |  |
| Remote symptomatic | 4 (2.9%) |  | 1 (6.7%) |  |  |
| Unknown | 37 (26.8%) |  | 7 (46.7%) |  |  |
| Acute cerebral event | 15 (10.9%) |  | 0 (0.0%) |  |  |
| Tumor | 6 (4.3%) |  | 0 (0.0%) |  |  |
| Other | 2 (1.4%) |  | 1 (6.7%) |  |  |
| **Goal of surgery** |  | 145 |  | 16 | 0.071 |
| Non-resective | 52 (35.9%) |  | 10 (62.5%) |  |  |
| Resective | 93 (64.1%) |  | 6 (37.5%) |  |  |

Abbreviations: SE, status epilepticus; FTBTC, focal to bilateral tonic-clonic; FIAS, focal impaired awareness; NCSE, non-convulsive status epilepticus; MRI, magnetic resonance imaging; EEG, electroencephalogram; mRS, modified Rankin Scale; RSE, refractory status epilepticus; SRSE, super-refractory status epilepticus.

All categorical variables are displayed in frequency (valid percentage) unless otherwise specified.

^a^ Chi-squared test or Fisher exact test for categorical variables and Welch’s ANOVA or Mann-Whitney U test for numeric variables.

* Statistically significant

Table S4. Clinical characteristics of patients stratified by their mRS at last follow-up.

|  | **Favorable mRS at last follow-up** | | | | |
| --- | --- | --- | --- | --- | --- |
| **Characteristic** | **Yes**  **N = 35** | **N** | **No**  **N = 44** | **N** | **p value** |
| **Sex (F)** | 10 (29.4%) | 34 | 24 (54.5%) | 44 | 0.047* |
| **Age at SE onset (years)** |  | 34 |  |  | 0.86 |
| Median (IQR) | 20.0 (7.5-37.8) |  | 22.0 (6.0-35.3) | 44 |  |
| Mean (SD) | 24.4 (20.0) |  | 25.0 (21.2) |  |  |
| Range | 0.0-67.0 |  | 0.1-68.0 |  |  |
| **New-onset seizures** | 14 (40.0%) | 35 | 20 (45.5%) | 44 | 0.80 |
| **History of SE** | 3 (9.4%) | 32 | 10 (26.3%) | 38 | 0.13 |
| **SRSE** | 27 (77.1%) | 35 | 43 (97.7%) | 44 | 0.0088* |
| **Time in SE prior to surgery (days)** |  | 25 |  | 39 | 0.25 |
| Median (IQR) | 19.0 (12.0-42.0) |  | 27.0 (14.5-60.0) |  |  |
| Mean (SD) | 178.9 (687.5) |  | 86.0 (237.0) |  |  |
| Range | 5.0-3,468.0 |  | 5.0-1,460.0 |  |  |
| **SE semiology classification** |  | 35 |  | 43 | 0.37 |
| FTBTC SE | 9 (25.7%) |  | 13 (30.2%) |  |  |
| FIAS SE | 11 (31.4%) |  | 9 (20.9%) |  |  |
| Focal aware SE | 10 (28.6%) |  | 9 (20.9%) |  |  |
| Generalized NCSE | 2 (5.7%) |  | 1 (2.3%) |  |  |
| Generalized myoclonic SE | 0 (0.0%) |  | 3 (7.0%) |  |  |
| Generalized convulsive SE | 3 (8.6%) |  | 8 (18.6%) |  |  |
| **EEG Localization** |  | 35 |  | 43 | 0.29 |
| Focal | 29 (82.9%) |  | 31 (72.1%) |  |  |
| Generalized | 6 (17.1%) |  | 12 (27.9%) |  |  |
| **Focal slowing** | 6 (21.4%) | 28 | 2 (6.5%) | 31 | 0.13 |
| **Diffuse slowing** | 2 (7.1%) | 28 | 8 (25.8%) | 31 | 0.084 |
| **MRI finding** |  | 31 |  | 35 | 0.86 |
| Focal | 16 (51.6%) |  | 16 (45.7%) |  |  |
| Diffuse | 8 (25.8%) |  | 11 (31.4%) |  |  |
| Negative | 7 (22.6%) |  | 8 (22.9%) |  |  |
| **Concordant MRI-EEG findings** | 12 (42.9%) | 28 | 12 (41.4%) | 29 | >0.99 |
| **Etiology** |  | 35 |  | 44 | 0.72 |
| Known epilepsy | 11 (31.4%) |  | 19 (43.2%) |  |  |
| Remote symptomatic | 0 (0.0%) |  | 2 (4.5%) |  |  |
| Unknown | 14 (40.0%) |  | 14 (31.8%) |  |  |
| Acute cerebral event | 7 (20.0%) |  | 6 (13.6%) |  |  |
| Tumor | 2 (5.7%) |  | 2 (4.5%) |  |  |
| Other | 1 (2.9%) |  | 1 (2.3%) |  |  |
| **Goal of surgery** |  | 35 |  | 44 | >0.99 |
| Non-resective | 15 (42.9%) |  | 19 (43.2%) |  |  |
| Resective | 20 (57.1%) |  | 25 (56.8%) |  |  |

Abbreviations: SE, status epilepticus; FTBTC, focal to bilateral tonic-clonic; FIAS, focal impaired awareness; NCSE, non-convulsive status epilepticus; MRI, magnetic resonance imaging; EEG, electroencephalogram; mRS, modified Rankin Scale; RSE, refractory status epilepticus; SRSE, super-refractory status epilepticus.

All categorical variables are displayed in frequency (valid percentage) unless otherwise specified.

^a^ Chi-squared test or Fisher exact test for categorical variables and Welch’s ANOVA or Mann-Whitney U test for numeric variables.

* Statistically significant

Table S5. Clinical characteristics of patients stratified by their SE outcome following initial intervention.

|  | **Epilepsy-related death** | | | | |
| --- | --- | --- | --- | --- | --- |
| **Characteristic** | Yes  N = 9 | N | No  N = 152 | N | p value |
| **Sex (F)** | 5 (55.6%) | 9 | 56 (45.9%) | 122 | 0.83 |
| **Age at SE onset (years)** |  | 9 |  | 151 | 0.0071* |
| Median (IQR) | 53.0 (25.0-57.0) |  | 13.8 (5.0-24.5) |  |  |
| Mean (SD) | 39.3 (22.7) |  | 18.0 (17.3) |  |  |
| Range | 0.6-65.0 |  | 0.0-68.0 |  |  |
| **New-onset seizures** | 4 (44.4%) | 9 | 38 (25.0%) | 152 | 0.24 |
| **History of SE** | 2 (33.3%) | 6 | 19 (16.5%) | 115 | 0.28 |
| **SRSE** | 9 (100.0%) | 9 | 127 (87.6%) | 145 | 0.60 |
| **Time in SE prior to surgery (days)** |  | 9 |  | 123 | 0.32 |
| Median (IQR) | 33.0 (16.0-67.0) |  | 21.0 (13.0-42.0) |  |  |
| Mean (SD) | 45.4 (33.1) |  | 115.4 (411.1) |  |  |
| Range | 5.0-90.0 |  | 2.0-3468.0 |  |  |
| **SE semiology classification** |  | 9 |  | 150 | 0.047* |
| FTBTC SE | 3 (33.3%) |  | 33 (22.0%) |  |  |
| FIAS SE | 1 (11.1%) |  | 39 (26.0%) |  |  |
| Focal aware SE | 1 (11.1%) |  | 53 (35.3%) |  |  |
| Generalized NCSE | 0 (0.0%) |  | 8 (5.3%) |  |  |
| Generalized myoclonic SE | 0 (0.0%) |  | 4 (2.7%) |  |  |
| Generalized convulsive SE | 4 (44.4%) |  | 13 (8.7%) |  |  |
| **EEG Localization** |  | 9 |  | 150 | 0.081 |
| Focal | 5 (55.6%) |  | 122 (81.3%) |  |  |
| Generalized | 4 (44.4%) |  | 28 (18.7%) |  |  |
| **Focal slowing** | 0 (0.0%) | 4 | 14 (10.9%) | 128 | >0.99 |
| **Diffuse slowing** | 0 (0.0%) | 4 | 19 (14.8%) | 128 | >0.99 |
| **MRI finding** |  | 6 |  | 129 | 0.73 |
| Focal | 3 (50.0%) |  | 72 (55.8%) |  |  |
| Diffuse | 1 (16.7%) |  | 33 (25.6%) |  |  |
| Negative | 2 (33.3%) |  | 24 (18.6%) |  |  |
| **Concordant MRI-EEG findings** | 1 (33.3%) | 3 | 56 (46.3%) | 121 | >0.99 |
| **Etiology** |  | 9 |  | 144 | 0.39 |
| Known epilepsy | 3 (33.3%) |  | 77 (53.5%) |  |  |
| Remote symptomatic | 1 (11.1%) |  | 4 (2.8%) |  |  |
| Unknown | 4 (44.4%) |  | 40 (27.8%) |  |  |
| Acute cerebral event | 1 (11.1%) |  | 14 (9.7%) |  |  |
| Tumor | 0 (0.0%) |  | 6 (4.2%) |  |  |
| Other | 0 (0.0%) |  | 3 (2.1%) |  |  |
| **Goal of surgery** |  | 9 |  | 152 | 0.31 |
| Non-resective | 5 (55.6%) |  | 57 (37.5%) |  |  |
| Resective | 4 (44.4%) |  | 95 (62.5%) |  |  |

Abbreviations: SE, status epilepticus; FTBTC, focal to bilateral tonic-clonic; FIAS, focal impaired awareness; NCSE, non-convulsive status epilepticus; MRI, magnetic resonance imaging; EEG, electroencephalogram; mRS, modified Rankin Scale; RSE, refractory status epilepticus; SRSE, super-refractory status epilepticus.

All categorical variables are displayed in frequency (valid percentage) unless otherwise specified.

^a^ Chi-squared test or Fisher exact test for categorical variables and Welch’s ANOVA or Mann-Whitney U test for numeric variables.

* Statistically significant

Table S6. Breakdown of surgical techniques and outcomes in the resection subgroup.

| **Characteristic** | **Focal resection**  N = 50 | **Lobectomy**  N = 12 | **Multilobar resection**  N = 2 | **Anterior quadrantotomy**  N = 1 | **Posterior quadrantotomy**  N = 4 | **Hemispheric surgery**  N = 26 | **MST +**  N = 4 |
| --- | --- | --- | --- | --- | --- | --- | --- |
| **SE cessation after initial intervention** | 45  (90.0%) | 11  (91.7%) | 2  (100.0%) | 1  (100.0%) | 4  (100.0%) | 26  (100.0%) | 4 (100.0%) |
| **Time to SE cessation (days)** |  |  |  |  |  |  |  |
| Median (IQR) | 0.0  (0.0 -0.0) | 0.0  (0.0 – 0.0) | 0.0  (NA) | NA | 0.0  (NA) | 0.0  (0.0 – 0.0) | 0.0  (0.0 – 0.0) |
| Range | 0.0 – 76.0 | 0.0 – 0.0 | 0.0 – 65.0 | NA | 0.0 – 0.0 | 0.0 – 0.0 | 0.0 – 0.0 |
| **Seizure freedom** | 32 (65.3%) | 6  (54.5%) | 1  (50.0%) | 1  (100.0%) | 2  (50.0%) | 22  (84.6%) | 3 (75.0%) |
| **Death** | 4  (8.0%) | 1  (8.3%) | 0  (0.0%) | 0  (0.0%) | 0  (0.0%) | 1 (3.8%) | 0  (0.0%) |
| **Epilepsy/SE-related death** | 3 (6.0%) | 1 (8.3%) | 0 (0.0%) | 0 (0.0%) | 0 (0.0%) | 0 (0.0%) | 0 (0.0%) |
| **Favorable mRS** | 14 (48.3%) | 2  (33.3%) | 1  (50.0%) | NA | NA | 2  (28.6%) | 1 (100.0%) |

Table S7. Stimulation parameters at stimulation onset and SE cessation, titration periods and repones to treatment in patients that underwent neuromodulation.

| Study ID | Technique | Stimulation parameter at stimulation onset | Stimulation parameters at SE cessation or final stimulation parameter tried | Stimulation parameter adjustment period (days) | SE cessation |
| --- | --- | --- | --- | --- | --- |
| Lee et al. 2016 | DBS | 5V, 145 Hz, 90 microseconds, bipolar, continuous | 8V, 145 Hz, 90 microseconds, bipolar, continuous | 0 | Yes |
| Yuan et al. 2019 | DBS | 1V, 130 Hz, 90 microseconds | 1V, 130 Hz, 90 microseconds | 0 | Yes |
| Stavropoulos et al. 2019 | DBS | 1.5mA, 130 Hz, 90 microseconds, bipolar, | 3V, 60 Hz, 300 microseconds, bipolar | 3 | Yes |
| Imbach et al. 2019 | DBS | 1V, 145 Hz, 90 microseconds, bipolar, continuous | 3V, 145 Hz, 90 microseconds, monopolar, continuous | 1 | Yes |
| Lehtimaki et al. 2016 | DBS | Not available | 7 V, 180 Hz, 150 microseconds, bipolar continuous | 14 | Yes |
| Franzini et al. 2008 | DBS | Not available | 2 V, 100 Hz, 90 microseconds, monopolar, continuous | NA | Yes |
| Valentin et al. 2012 | DBS | Not available | 5 V, 6 Hz, 90 microseconds, bipolar, continuous | 20 | Yes |
| Sa et al. 2019 | DBS | 4mA, 130 Hz, 90 microseconds, bipolar | 2 mA, 6 Hz, 450 microseconds, bipolar | 7 | Yes |
| Sa et al. 2019 | DBS | 2 mA, 130 Hz, 90 microseconds, bipolar | 2 mA, 6 Hz, 450 microseconds, bipolar | 4 | No |
| Courtin et al. 2022 | DBS | Not available | 3 mA, 130 Hz, 60 microseconds, bipolar | NA | No |
| Mamaril-Davis et al. 2022 | RNS | Not available | 6 mA, R precentral gyrus: 200 Hz R postcentral gyrus: 5 Hz, 160 microseconds, burst duration: R precentral gyrus (400 milliseconds) R postcentral gyrus (5000 milliseconds) | NA | Yes |
| Ernst et al. 2019 | RNS | 5 mA, 100 Hz, 120 mircroseconds, burst duration: 500 milliseconds | 10 mA, 5 Hz, 200 mircroseconds, burst duration: 3000 milliseconds | 14 | Yes |
| Graley et al. 2021 | RNS | Not available | 3.6 mA, 200 Hz, 160 microseconds, burst duration | NA | Yes |
| Yang et al. 2021 | RNS | 1 mA, 200 Hz, 160 microseconds, burst duration: 100 milliseconds | 1 mA, 200 Hz, 160 microseconds, burst duration: 100 milliseconds | 0 | Yes |
| Chang et al. 2019 | SCS | 1 V, 5 Hz, 150 microseconds | 1 V, 5 Hz, 150 microseconds | 0 | Yes |
| Chang et al. 2019 | SCS | 1 V, 5 Hz, 90 microseconds | 1 V, 5 Hz, 90 microseconds | NA | Yes |
| Chang et al. 2019 | SCS | 2 V, 3 Hz, 90 microseconds | 2 V, 3 Hz, 90 microseconds | NA | Yes |
| Schrader et al. 2006 | SCS | Not available | 16 mA, 0.5 Hz, 500 microseconds | 7 | No |
| Valentin et al. 2015 | SCS | Not available | 3 mA, 60 H, 450 microseconds | 4 | Yes |
| Valentin et al. 2015 | SCS | Not available | 3 mA, 130 Hz, 450 microseconds | 5 | Yes |
| Alsaadi et al. 2015 | VNS | Not available | 2.5 mA, DC 10% | 4 | Yes |
| Hect et al. 2022 | VNS | Not available | 2.5 mA, DC 58% | NA | No |
| Kurukumbi et al. 2019 | VNS | 1.5 mA, 30 Hz, 500 microseconds, DC 16% | 1.5 mA, 30 Hz, 500 microseconds, DC 16% | 0 | Yes |
| Specchio et al. 2020 | VNS | 0.25 mA, 30 Hz, 500 microseconds, DC 10% | 1.75 mA, 30 Hz, 500 microseconds, DC 10% | 7 | Yes |
| Specchio et al. 2020 | VNS | 0.25 mA, 30 Hz, 500 microseconds, DC 10% | 1 mA, 30 Hz, 500 microseconds, DC 10% | 10 | Yes |
| Al-Attas et al. 2022 | VNS | Not available | Not available | NA | Yes |
| De Herdt et al. 2009 | VNS | 0.25 mA, 30 Hz, 500 microseconds, DC 10% | 1.5 mA, 30 Hz, 500 microseconds, DC 10% | 4 | Yes |
| Mostacci et al. 2019 | VNS | 0.125 mA, 30 Hz, 250 microseconds, DC 10% | 1.75 mA, 30 Hz, 500 microseconds, DC 28% | 5 | Yes |
| Luo et al. 2022 | VNS | 0.2 mA, 30 Hz, 500 microseconds, DC 10% | 3 mA, 30 Hz, 750 microseconds, DC 19% | 27 | Yes |
| Yazdi et al. 2016 | VNS | 1.5 mA, 30 Hz, 500 microseconds, 29% DC | 1.5 mA, 30 Hz, 500 microseconds, DC 29% | 0 | Yes |
| Espino et al. 2022 | VNS | 0.25 mA, 20 Hz, 250 microseconds, DC 16% | 1.75 mA, 20 Hz, 250 microseconds, DC 16% | 7 | Yes |
| Braakman et al. 2018 | VNS | 0.25 mA, 30 hz, 500 microseconds, DC 28% | 0.25 mA, 30 hz, 500 microseconds, DC 28% | 0 | Yes |
| Dhaliwal et al. 2020 | VNS | 0.25 mA, 30 hz, 250 microseconds, DC 10% | 0.75 mA, 30 Hz, 250 microseconds, DC 12% | 7 | Yes |
| O'Neill et al. 2011 | VNS | 1 mA, 25 Hz, 250 microseconds, DC 39% | 1 mA, 25 Hz, 250 microseconds, DC 45% | 1 | Yes |
| Patwardhan et al. 2005 | VNS | 0.25 mA, 20 Hz, 250 microseconds, DC 10% | 1 mA, 20 Hz, 250 microseconds, DC 17% | NA | Yes |
| Winston et al. 2000 | VNS | 0.25 mA, 30 Hz, 500 microseconds, DC 6% | 0.25 mA, 30 Hz, 500 microseconds, DC 6% | 2 | Yes |
| Zamponi et al. 2008 | VNS | 0.25 mA DC 10% | 1.75 mA, DC 10% | 13 | Yes |
| Zamponi et al. 2008 | VNS | 0.25 mA DC 10% | 2 mA, DC 10% | NA | Yes |
| De Bendictis et al. 2013 | VNS | Not available | 1.5 mA | 21 | Yes |
| De Bendictis et al. 2013 | VNS | Not available | 2.5 mA | 60 | Yes |
| De Bendictis et al. 2013 | VNS | Not available | 2.5 mA | 21 | Yes |
| De Bendictis et al. 2013 | VNS | Not available | 1.75 mA | 30 | Yes |
| Rodriguez-Villar et al. 2023 | VNS | Not available | 1.5-2mA | 3 | Yes |
| Jindal et al. 2023 | VNS | 0.25 mA, 30 Hz, 500 microseconds, DC 10% | 1 mA, 30 Hz, 500 microseconds, DC 10% | 7 | Yes |
| Mehboob et al. 2023 | VNS | Not available | 2 mA, 58% DC | 21 | Yes |
| Melinda Furlanis et al. 2023 | VNS | 0.25 mA, 500 microseconds, DC 10% | 1 mA, 500 microseconds, DC 10% | 4 | Yes |
| Melinda Furlanis et al. 2023 | VNS | 0.25 mA, 500 microseconds, DC 10% | 1 mA, 500 microseconds, DC 10% | 5 | Yes |
| Melinda Furlanis et al. 2023 | VNS | 0.25 mA, 500 microseconds, DC 10% | 1 mA, 250 microseconds, DC 10% | 1.5 | Yes |
| Melinda Furlanis et al. 2023 | VNS | 0.25 mA, 500 microseconds, DC 10% | 1 mA, 250 microseconds, DC 10% | NA | No |
| Melinda Furlanis et al. 2023 | VNS | 0.25 mA, 30 hz, 250 microseconds, DC 10% | 2.25 mA, 30 Hz, 250 microseconds, 16% DC | 8 | Yes |
| Melinda Furlanis et al. 2023 | VNS | Not available | 2 mA, 20 Hz, 250 microseconds, 15% DC | NA | Yes |
| Melinda Furlanis et al. 2023 | VNS | Not available | N/A | NA | No |
